# Haplotype-resolved DNA methylation at the *APOE* locus identifies allele-specific epigenetic signatures relevant to Alzheimer’s disease risk

**DOI:** 10.1038/s44400-026-00094-8

**Published:** 2026-06-19

**Authors:** Rylee M. Genner, Melissa Meredith, Kensuke Daida, Abraham Moller, Cory Weller, Alexis Ayuketah, Pilar Alvarez Jerez, Stuart Akeson, Laksh Malik, Breeana Baker, Cedric Kouam, Kimberly Paquette, Adam Catching, Sarah Bromberek, Fangle Hu, Xylena Reed, Stefano Marenco, Pavan Auluck, Ajeet Mandal, Benedict Paten, J. Raphael Gibbs, Miten Jain, Mark R. Cookson, Andrew B. Singleton, Mike Nalls, Cornelis Blauwendraat, Kimberley J. Billingsley

**Affiliations:** 1 Center for Alzheimer’s and Related Dementias, National Institute on Aging and National Institute of Neurological Disorders and Stroke, National Institutes of Health, Bethesda, MD USA; 2 Department of Biology, Johns Hopkins University, Baltimore, MD USA; 3 UC Santa Cruz Genomics Institute, Santa Cruz, CA USA; 4 DataTecnica LLC, Washington, DC USA; 5 Laboratory of Neurogenetics, National Institute on Aging, National Institutes of Health, Bethesda, MD USA; 6 Department of Neurodegenerative Disease, UCL Queen Square Institute of Neurology, University College London, London, UK; 7 Global Parkinson’s Genetics Program (GP2), Chevy Chase, MD USA; 8 Department of Bioengineering, Northeastern University, Boston, MA USA; 9 Human Brain Collection Core, Division of Intramural Research, National Institute of Mental Health, NIH, Bethesda, MD USA

**Keywords:** Genetics, Molecular biology, Neuroscience

## Abstract

The *APOE* gene encodes a lipid transport protein central to Alzheimer’s disease (AD) pathogenesis. Three common alleles—ε2 (rs7412(C > T)), ε3 (reference), and ε4 (rs429358(T > C))—arise from two coding variants in exon 4 and confer distinct AD risk profiles, with ε4 increasing risk and ε2 being protective. The ε3-linked *APOE* variant rs769455[T] has also been associated with increased AD risk among individuals of African ancestry who also carry the *APOE* ε4 allele. Determining how genetic variation influences CpG methylation requires methQTL-type analyses, but conventional bisulfite and array-based approaches offer limited resolution for distinguishing allele-specific effects. Here, we use high-accuracy long-read sequencing to generate haplotype-resolved methylation profiles across the *APOE* locus in 332 postmortem brain tissue samples from ancestrally diverse cohorts, including 201 samples from individuals of European ancestry and 131 samples from individuals of African and African admixed ancestry. Treating each haplotype as an independent observation, OLS regression identified 18 novel differentially methylated CpG sites associated with ε2, ε4, and rs769455[T] across the *APOE* locus (*TOMM40, APOE, APOC1*, and *APOC4*-*APOC2* genes). These findings reveal distinct allele-specific methylation signatures and demonstrate the utility of long-read sequencing for resolving epigenetic variation relevant to AD risk.

## Introduction

The *Apolipoprotein E* (*APOE*) gene, located on chromosome 19, encodes a lipid transport protein essential for neuronal development, maintenance, and repair^1–3^. *APOE* plays a critical role in the central nervous and cardiovascular systems and is a major genetic determinant of Alzheimer’s disease (AD) risk^4,5^. Two single nucleotide variants (SNVs) in *APOE* exon 4 define the ε2 (rs7412(C > T)), ε3 (reference), and ε4 (rs429358(T > C)) alleles that modulate AD susceptibility. The ε4 allele is the strongest common genetic risk factor for late-onset AD (LOAD), increasing disease risk in a dose-dependent manner estimated to be 2- to 12-fold compared to the reference ε3 allele^5–8^, while the ε2 allele is shown to reduce LOAD risk in a dose-dependent manner^9^. An *APOE*-ε3-linked missense variant rs769455(C > T) was recently identified as a potential population-specific AD risk factor in individuals of African ancestry who carry both the rs769455[T] allele and the ε4 allele^10,11^. The roles that epigenetic modifications play in disease development cannot be fully understood without considering the population specificities of methylation, and the discovery of ancestry-associated variants like rs769455[T] underscores the need for studying *APOE* genetic and epigenetic variation across genetic ancestries.

Epigenetic modifications like DNA methylation can also influence *APOE* expression and have been implicated in AD pathogenesis^12,13^. 5-methylcytosine (5mC) refers to the transient addition of a methyl group to the fifth carbon position of a cytosine residue. This occurs most often at cytosines that are immediately adjacent to guanines, commonly called cytosine-phosphate-guanine (CpG) sites, which can be condensed within regulatory regions called CpG islands^14–16^. The *APOE* gene cluster describes a genomic region in strong linkage disequilibrium that spans the *TOMM40, APOE, APOC1*, and *APOC4-APOC2* genes^17–21^. This cluster was first defined by Cervantes et al. (2011) and contains CpG islands and other regulatory regions that may be modulating neurodegeneration by regulating gene expression and lipid metabolism in the brain^21^. Previous studies using short-read sequencing (SRS) and bisulfite sequencing have reported differentially methylated CpG sites associated with the ε2 and ε4 alleles in this cluster^13,22–25^. However, these approaches face several limitations: (i) bisulfite conversion can degrade DNA^26–28^; (ii) array-based methods, such as the Illumina Infinium HumanMethylation450 and 850 BeadChip arrays, provide incomplete CpG site coverage^29^; and (iii) SRS generates fragmented reads that limit phasing abilities and can hinder the analysis of repetitive or structurally complex regions of the genome^30–32^. As a result, prior studies have primarily examined associations between *APOE* genotype and total methylation levels (averaged across both alleles) without resolving allele-specific epigenetic differences (Supplementary Table S1). Long-read sequencing (LRS) can overcome these limitations by directly detecting genome-wide nucleotide modifications at single-nucleotide resolution. Oxford Nanopore Technologies (ONT) LRS, in particular, enables phased haplotype-resolved methylation analysis of long DNA fragments (10 kb – 1 Mb) along with direct methylation detection^33–35^, allowing more precise characterization of epigenetic variation in genomic regions relevant to neurodegenerative disease.

We leveraged publicly available LRS data from 332 postmortem frontal cortex samples from individuals of European (EUR) and African and African admixed (AFR) ancestry that were sequenced as part of the NIH Center for Alzheimer’s and Related Dementias (CARD) Long-Read Sequencing Initiative (https://card.nih.gov/research-programs/long-read-sequencing). We examined allele-specific DNA methylation associated with three *APOE* variants (the ε2 and ε4 alleles (EUR and AFR cohorts) and the rs769455[T] SNV (AFR cohort only)), across a 47,587 bp region encompassing the full *APOE* gene cluster. We also obtained cell-type proportion estimates generated from single-nucleus RNA-seq data from adjacent cortical tissue to evaluate the potential influence of cellular composition on methylation levels.

Our study advances the understanding of the epigenetic mechanisms linking *APOE* variation to AD risk alleles by resolving allele-specific methylation patterns in an ancestrally diverse dataset; however, future functional studies will be important for determining whether these methylation differences affect *APOE* gene regulation or are associated with other pathways relevant to AD.

## Results

### Comprehensive resolution of haplotypes and allele-specific methylation through long-read sequencing

We leveraged publicly available datasets from the CARD Long-read Sequencing Initiative, comprising whole-genome sequencing (WGS) ONT sequencing data from 332 postmortem brain samples from individuals with no known neurological diseases across the NABEC (*n* = 201) and HBCC (*n* = 131) cohorts^36^. The average age of the samples used in the analysis was 52.37 years for NABEC and 45.19 years for HBCC. Female donors comprised 35.6% of the NABEC samples and 38.9% of the HBCC samples. A genetic ancestry analysis determined that the NABEC samples were most genetically similar to reference samples from European populations (EUR), while the HBCC samples were most genetically similar to reference samples from African and African admixed populations (AFR) (Supplementary Table S2). Two AD-associated alleles (ε2 and ε4) and one AD-associated variant (rs769455[T]) located in *APOE* exon 4 with minor allele frequency (MAF) > 0.01 were selected for the allele-specific methylation analysis (Table 1).

**Table 1:** Counts and minor allele frequencies (MAFs) of the *APOE* alleles and rs769455[T] variant included in the analysis

APOE allele	rs429358	rs7412	Region	hg38 coord.	Alleles (MAF 1st)	AD assoc.	EUR MAF	AFR MAF	NABEC (EUR) MAF	HBCC (AFR) MAF
ε2	*T*	*T*	APOE exon 4 CGI	chr19: 44908822	rs7412 C > T	Protective	0.067	0.11	0.11	0.081
ε4	*C*	*C*	APOE exon 4 CGI	chr19: 44908684	rs429538 T > C	Pathogenic	0.15	0.22	0.15	0.21
rs769455	--	--	APOE exon 4 CGI	chr19: 44908783	rs769455 C > T	Pathogenic	0	0.021	0	0.023
								**Total haplotypes:**	402	262

EUR (European) and AFR (African and African admixed) MAFs were taken from Gnomad v4.1.0 using the following sample totals: ε2, ε4 and rs769455[T] European (EUR) totals = 89,919, 174,295, and 74 respectively and ε2, ε4 and rs769455[T] African and African American (AFR) sample totals = 7729, 16,245, and 1549, respectively. Allele counts from the NABEC and HBCC cohorts were used to calculate variant frequencies.

**Fig. 1:**
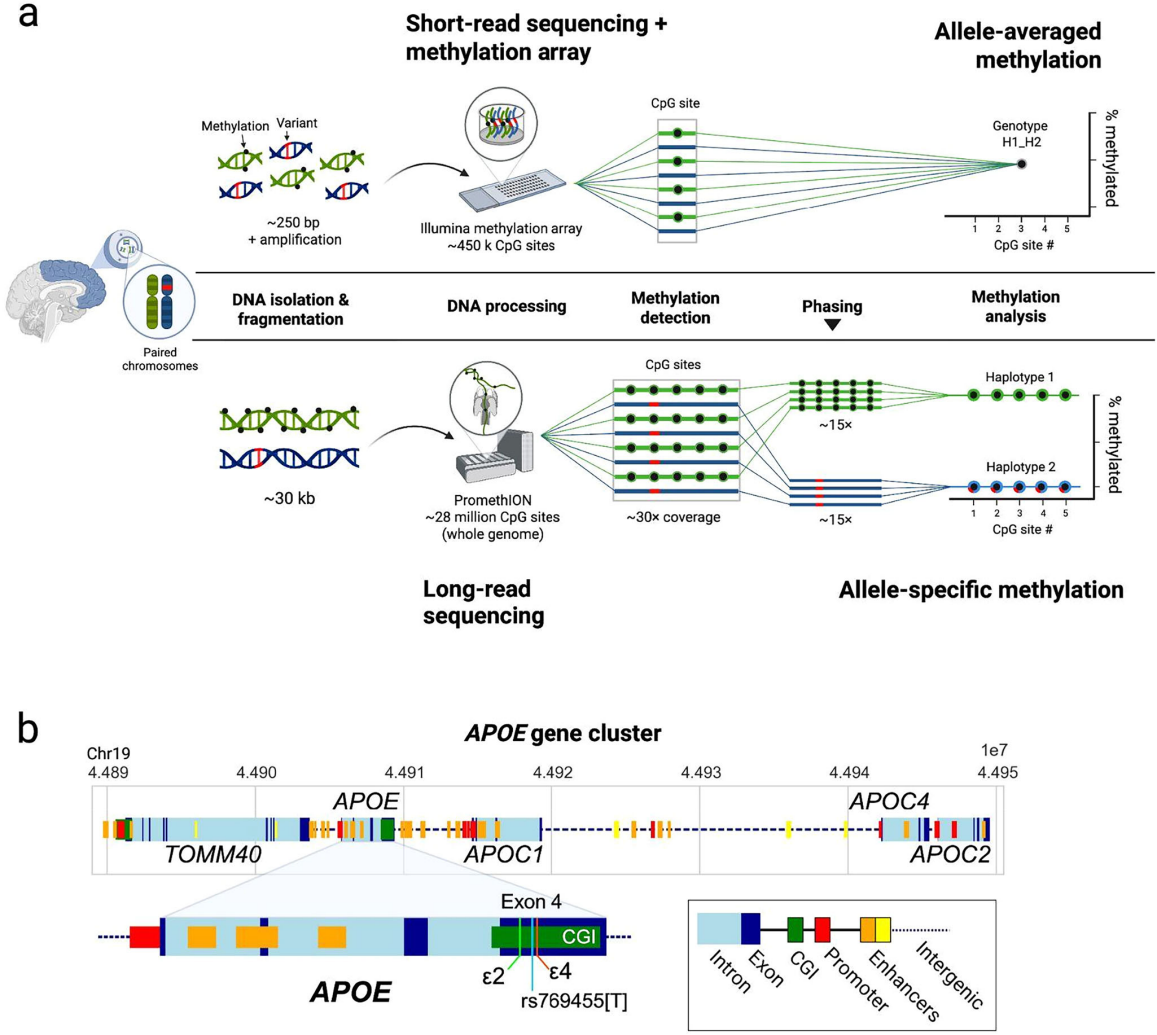
Comparison of methylation detection methods and schematic of APOE gene cluster. **a** Overview of the Illumina methylation array-based pipeline used to detect methylation levels in unphased genomic data (top) and the long-read sequencing pipeline used to detect allele-specific methylation differences (bottom). **b** Schematic of the *APOE* gene cluster, highlighting the *APOE* gene and the locations of the ε2 and ε4 alleles and rs769455[T] variant within the *APOE* exon 4 CpG island.

Allele-specific differential methylation profiling was performed at the *APOE* gene cluster (chr19:44889556–44953378, hg38), which encompasses the *TOMM40*, *APOE*, *APOC1*, and *APOC4-APOC2* genes (Fig. 1a). After phasing, the mean haplotype-specific coverage across this region was 12× (read N50 = 28.1 kb) in NABEC and 10.6× (read N50 = 25.6 kb) in HBCC samples (Supplementary Table S3). As expected, haplotype-level coverage was approximately half that of total diploid coverage. A schematic of the pipeline used to generate the haplotype-resolved variant calls and CpG methylation profiles^34^ along with representative graphs illustrating phased and unphased methylation frequency output is shown in Fig. 1b.

**Fig. 2:**
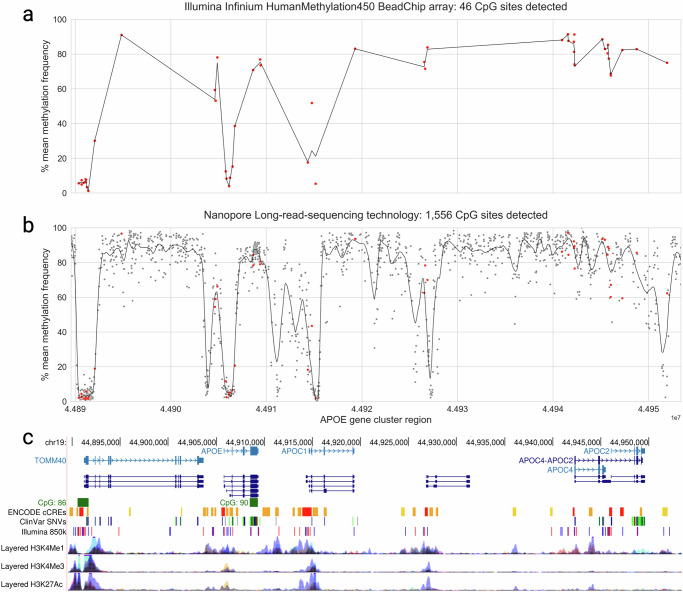
Comparison of CpG sites detected in the *APOE* gene cluster region by the Illumina Infinium HumanMethylation450 BeadChip array and ONT’s long-read sequencing technology. Plots depict the mean methylation frequencies of CpG sites across samples detected in the *APOE* gene cluster (*TOMM40, APOE, APOC1, and APOC4*-*APOC2*; hg38 chr19:44889556-44953378). **a** Mean methylation of 46 CpG sites in 697 samples from the Religious Orders Study/Memory and Aging Project, detected by the Illumina Infinium HumanMethylation450 BeadChip array. These samples came from a mix of AD and control individuals of European ancestry. **b** Mean methylation of 1,556 CpG sites in 205 samples from the North American Brain Expression Cohort detected by ONT’s PromethION long-read sequencing data as part of the NIH CARD Long Read Initiative. CpG sites also detected by the Illumina Infinium HumanMethylation450 BeadChip array are highlighted in red. **c**) UCSC Genome Browser tracks of the *APOE* gene cluster, displaying GENCODE V47 genes, RefSeq transcripts, CpG islands, ENCODE Candidate Cis-Regulatory Elements, ClinVar SNVs, Illumina Infinium HumanMethylation850 BeadChip array probes, and ENCODE histone marks (H3K4Me1, H3K4Me3, H3K27Ac). Tracks align with Fig. 2a, b coordinates.

To evaluate the comprehensiveness of long-read methylation profiling, we compared CpG detection in our ONT data to an Illumina Infinium HumanMethylation450 BeadChip (Illumina 450k) array of bulk brain tissue from the Religious Orders Study/Memory and Aging Project (ROSMAP) study—a large, well-characterized cohort of postmortem brain methylation data from aged individuals^37^. Long-read sequencing enabled higher-resolution methylation profiling of the *APOE* gene cluster and substantially increased the number of CpG sites detected compared to the Illumina 450k array. ONT WGS data from the NABEC samples (*n* = 201, EUR ancestry) identified 1556 CpG sites across the *APOE* gene cluster, whereas the Illumina 450k array data from ROSMAP samples (*n* = 697, EUR ancestry) detected only 46 CpG sites (Fig. 2a, b). One of the updated versions of this array is the Illumina Infinium HumanMethylation850 BeadChip array which measures methylation of over 850k CpG sites across the human genome. This array would have detected 76 CpG sites within the *APOE* gene cluster region, and these sites can be seen as blue and red lines along the “Illumina 850k” UCSC Genome track of Fig. 2c. We did not have access to sequencing data from frontal cortex brain tissue samples that had been sequenced with this upgraded array, which is why the Illumina Infinium HumanMethylation450 BeadChip array data was used in this comparison. The 46 CpG sites that were detected in both brain tissue cohorts by both ONT LRS and the Illumina 450k array (Fig. 2b, red points) had highly correlated methylation frequencies (Pearson r = 0.98) (Supplementary Fig. 1).

CpG sites detected by methylation arrays are largely restricted to promoter and exonic regions, whereas long-read profiling extends coverage to intronic, intergenic, and regulatory regions (Fig. 2c) to capture a broader methylation landscape. Overall, ONT LRS detected approximately 33× more CpG sites than the Illumina Infinium HumanMethylation450 BeadChip array, demonstrating its ability to detect methylation at sites that are not targeted by the conventional methods.

### Haplotype-resolved long-read sequencing refines phylogenetic structure

To evaluate the contribution of haplotype phasing to the resolution of genetic structure at the *APOE* locus, we inferred phylogenetic trees from both phased and unphased genomic sequences and visualized them as dendrograms. The phylogenetic trees were inferred with FastTree using an approximate maximum-likelihood framework and were rooted using the chimpanzee *APOE* ε4 sequence, which represents the ancestral *APOE* allele^38^. The unphased dendrogram demonstrated genotype-level separation but showed discontinuous and partially overlapping clustering of alleles (Supplementary Fig. 2A). In contrast, the haplotype-resolved data yielded distinct clades corresponding to the *APOE* ε2, ε3, and ε4 alleles, as well as a separate cluster of haplotypes harboring the rs769455[T] variant (Supplementary Fig. 2B). A total of 664 phased haplotypes were resolved across the NABEC and HBCC datasets. Because recombination has been reported within the broader *APOE* locus, these trees should be interpreted as heuristic groupings of haplotypes rather than strict genealogical histories.

Given that NABEC and HBCC samples were sequenced using different ONT chemistries (R9 and R10, respectively), we included one sample from the NABEC cohort that was sequenced with both chemistries^35^ to test whether clustering was driven by sequence differences or chemistry-specific artifacts. The *APOE* alleles in this sample clustered together, supporting the robustness of the observed phylogenetic separation (Supplementary Fig. 3).

### Allele-specific differentially methylated CpG sites in the APOE gene cluster region

**Fig. 3:**
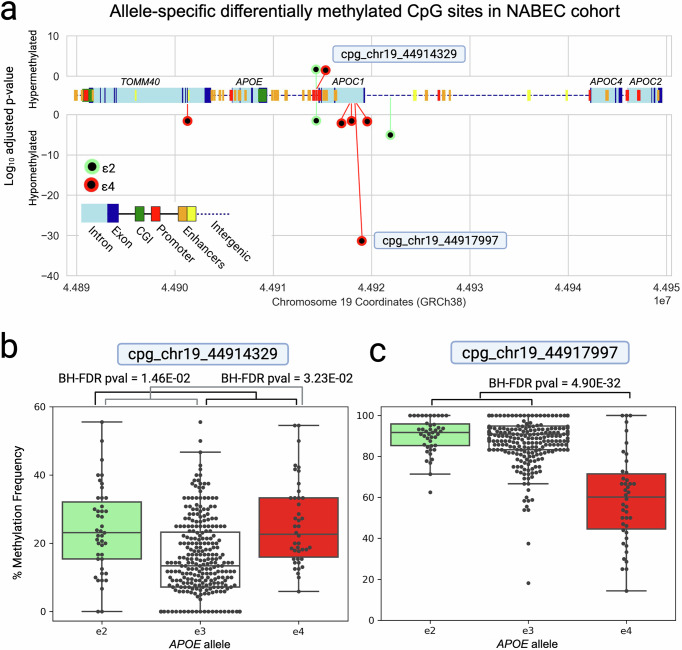
Differentially methylated CpG sites in the *APOE* locus associated with *APOE* ε2 and ε4 alleles in NABEC brain samples. **a** Association of *APOE* ε2 (green) and ε4 (red) alleles with methylation at DMCs within the *APOE* gene cluster. Each point represents a CpG site that was significantly differentially methylated (BH-FDR-corrected *p* value < 0.05). The y-axis shows the signed –log_10_(P) value, where positive values indicate hypermethylation and negative values indicate hypomethylation relative to haplotypes without the respective allele. CpG sites highlighted with labels are shown in the panels below. The middle x-axis provides a schematic of the *APOE* gene cluster region, and the bottom x-axis indicates chromosome 19 genomic coordinates (reference GRCh38). **b** Box-and-whisker plot showing methylation levels at CpG site cpg_chr19_44914329, stratified by *APOE* allele status. **c** Box-and-whisker plot showing methylation levels at CpG site cpg_chr19_44917997, stratified by *APOE* allele status.

An OLS linear regression model was used to identify differentially methylated CpG sites (DMCs) in the *APOE* gene cluster region of the NABEC and HBCC cohorts. In the NABEC cohort (*n* = 201; EUR ancestry), eight DMCs exhibited significant allele-specific methylation differences (BH-FDR corrected *p* value < 0.05). Three DMCs showed differential methylation between haplotypes carrying the ε2 allele relative to ε3 and ε4 (cpg_chr19_44914329, cpg_chr19_44914361, and cpg_chr19_44921919), and six DMCs showed differential methylation between haplotypes carrying the ε4 allele relative to ε2 and ε3 (cpg_chr19_44901266, cpg_chr19_44914329, cpg_chr19_44917921, cpg_chr19_44917959, cpg_chr19_44917997, and cpg_chr19_44918064). One site, cpg_chr19_44914329, was significant in both the ε2 and ε4 comparisons. These sites were distributed across functionally relevant regions: two were located within an uncharacterized single-exon gene ENSG00000280087.1 (cpg_chr19_44914329 and cpg_chr19_44914361), four overlapped a short interspersed nuclear element (SINE) within intron 3 of the *APOC1* gene (cpg_chr19_4491721, cpg_chr19_44917595, cpg_chr19_44917997, and cpg_chr19_44918064), and one was located in the intergenic region between *APOC1* and *APOE* (cpg_chr19_44921919) (Table 2; Fig. 3a).

**Table 2:** Differentially methylated CpG sites within the *APOE* cluster region in NABEC and HBCC cohorts associated with *APOE* ε2, ε4, or rs769455[T] (BH-FDR-corrected *p* value < 0.05).

Cohort	CpG site	Region	Allele	*N*	beta	SE	pval	BH-FDR corrected pval	Previously characterized
NABEC	cpg_chr19_44901266	TOMM40 exon 8 (out-of-frame exon) TOMM40 transcript ENST00000405636.6, TOMM40 transcript ENST00000252487.9, TOMM40 transcript ENST00000592434.5	e4	351	–5.49	1.38	9.03E–05	2.81E–02	no
NABEC	**cpg_chr19_44914329**	Uncharacterized single-exon gene ENSG00000280087 (transcript ENST00000623895.1) located between APOE and APOC1 and partly overlapping both genes, ENST00000588750.5 - APOC1 transcript variant 3, ENST00000588802.5 - APOC1 transcript variant 2, promoter-like signature, long terminal repeat element (ERV1 family), H3K27Ac mark enrichment	e2	361	8.32	1.96	2.81E–05	1.46E–02	yes; Shao et al. 2018, Panitch et al. 2024
NABEC	**cpg_chr19_44914329**		e4	361	8.07	2.08	1.25E–04	3.23E–02	no
NABEC	cpg_chr19_44914361	Uncharacterized single-exon gene ENSG00000280087 (transcript ENST00000623895.1) located between APOE and APOC1 and partly overlapping both genes, ENST00000588750.5 - APOC1 transcript variant 3, ENST00000588802.5 - APOC1 transcript variant 2, promoter-like signature, long terminal repeat element, H3K27Ac mark enrichment	e2	340	–8.91	2.2	6.27E–05	2.44E–02	no
NABEC	cpg_chr19_44917921	APOC1 intron 3, ENST00000588750.5 - APOC1 transcript variant 3, ENST00000588802.5 - APOC1 transcript variant 2, ENST00000592535.6 - APOC1 transcript variant 1, ENST00000592885.5 - APOC1 transcript variant 4, ENST00000589781.1 - APOC1 transcript variant (from HGNC APOC1), ENST00000586638.5 - APOC1 secreted transcript variant (from UniProt K7ELM9), short interspersed nuclear element (AluSx family)	e4	363	–7.96	1.77	9.67E–06	7.52E–03	no
NABEC	cpg_chr19_44917959	APOC1 intron 3, ENST00000588750.5 - APOC1 transcript variant 3, ENST00000588802.5 - APOC1 transcript variant 2, ENST00000592535.6 - APOC1 transcript variant 1, ENST00000592885.5 - APOC1 transcript variant 4, ENST00000589781.1 - APOC1 trancript variant (from HGNC APOC1), ENST00000586638.5 - APOC1 secreted transcript variant (from UniProt K7ELM9), short interspersed nuclear element (AluSx family)	e4	357	–7.51	1.89	8.51E–05	2.81E–02	no
NABEC	**cpg_chr19_44917997**	APOC1 intron 3, ENST00000588750.5 - APOC1 transcript variant 3, ENST00000588802.5 - APOC1 transcript variant 2, ENST00000592535.6 - APOC1 transcript variant 1, ENST00000592885.5 - APOC1 transcript variant 4, ENST00000589781.1 - APOC1 transcript variant (from HGNC APOC1), ENST00000586638.5 - APOC1 secreted transcript variant (from UniProt K7ELM9), dbSNP 155 rs12721046 G/A, short interspersed nuclear element (AluSg family)	e4	364	–29.74	2.13	3.15E–35	4.90E–32	no
NABEC	cpg_chr19_44918064	APOC1 intron 3, ENST00000588750.5 - APOC1 transcript variant 3, ENST00000588802.5 - APOC1 transcript variant 2, ENST00000592535.6 - APOC1 transcript variant 1, ENST00000592885.5 - APOC1 transcript variant 4, ENST00000589781.1 - APOC1 transcript variant (from HGNC APOC1), ENST00000586638.5 - APOC1 secreted transcript variant (from UniProt K7ELM9), short interspersed nuclear element (AluSg family)	e4	360	–8.79	2.12	4.24E–05	2.20E–02	no
NABEC	cpg_chr19_44921919	APOC1 - APOC4 intergenic, SVA_D retrotransposon	e2	344	–13.51	2.25	5.45E–09	4.24E–06	no
									no
HBCC	cpg_chr19_44890618	TOMM40 CpG island 86, proximal enhancer-like signature, RNA gene ENSG00000267282 / transcript ENST00000585408.2, H3K27Ac mark enrichment	rs769455[T]	238	1.15	0.29	1.01E–04	1.71E–02	no
HBCC	cpg_chr19_44890901	TOMM40 CpG island 86, TOMM40 promoter-like signature, RNA gene ENSG00000267282 / transcript ENST00000585408.2, H3K27Ac mark enrichment	rs769455[T]	249	2.29	0.6	1.94E–04	2.47E–02	no
HBCC	**cpg_chr19_44896082**	TOMM40 intron 5, distal enhancer-like signature, TOMM40 transcript ENST00000405636.6, TOMM40 transcript ENST00000252487.9, TOMM40 transcript ENST00000592434.5	e4	246	–4.56	0.97	4.36E–06	6.66E–03	no
HBCC	cpg_chr19_44896114	TOMM40 intron 5, TOMM40 transcript ENST00000405636.6, TOMM40 transcript ENST00000252487.9, TOMM40 transcript ENST00000592434.5	rs769455[T]	248	–4.93	1.29	1.64E–04	2.37E–02	no
HBCC	cpg_chr19_44896243	TOMM40 intron 5, TOMM40 transcript ENST00000405636.6, TOMM40 transcript ENST00000252487.9, TOMM40 transcript ENST00000592434.5	rs769455[T]	249	-12.5	3.08	6.72E–05	1.66E–02	no
HBCC	cpg_chr19_44899850	TOMM40 intron 5, TOMM40 transcript ENST00000405636.6, TOMM40 transcript ENST00000252487.9, TOMM40 transcript ENST00000592434.5 short interspersed nuclear element (Alu family)	rs769455[T]	248	–10.11	2.55	9.65E–05	1.71E–02	no
HBCC	**cpg_chr19_44904817**	TOMM40 - APOE intergenic, short interspersed nuclear element (MIR family), H3K27Ac mark enrichment	rs769455[T]	245	–30.24	5.49	1.03E–07	5.26E–05	no
HBCC	cpg_chr19_44915122	APOC1 intron 2, ENST00000588750.5 - APOC1 transcript variant 3, ENST00000588802.5 - APOC1 transcript variant 2, ENST00000592535.6 - APOC1 transcript variant 1, ENST00000592885.5 - APOC1 transcript variant 4, ENST00000589781.1 - APOC1 transcript variant (from HGNC APOC1), ENST00000586638.5 - APOC1 secreted transcript variant (from UniProt K7ELM9), proximal enhancer-like signature, H3K27Ac mark enrichment	rs769455[T]	250	1.64	0.35	4.07E–06	1.56E–03	no
HBCC	cpg_chr19_44915466	APOC1 intron 2, ENST00000588750.5 - APOC1 transcript variant 3, ENST00000588802.5 - APOC1 transcript variant 2, ENST00000592535.6 - APOC1 transcript variant 1, ENST00000592885.5 - APOC1 transcript variant 4, ENST00000589781.1 - APOC1 transcript variant (from HGNC APOC1), ENST00000586638.5 - APOC1 secreted transcript variant (from UniProt K7ELM9), proximal enhancer-like signature, short interspersed nuclear element (AluYc family), H3K27Ac mark enrichment	rs769455[T]	252	2.87	0.7	6.23E–05	1.66E–02	no
HBCC	cpg_chr19_44931155	ENSG00000291128.2 - APOC1 pseudogene 1 UTR exon 4 / ENST00000701959.2 - APOC1 pseudogene transcript, ENSG00000291128.2 - APOC1 pseudogene 1 UTR exon 3 / ENST00000574565.2 - APOC1 pseudogene 1 transcript variant 1	rs769455[T]	249	–9.01	2.23	7.58E–05	1.66E–02	no
HBCC	cpg_chr19_44941331	APOC1-APOC4 intergenic, short interspersed nuclear element (AluY family)	rs769455[T]	251	–5.4	1.41	1.70E–04	2.37E–02	no

**Fig. 4:**
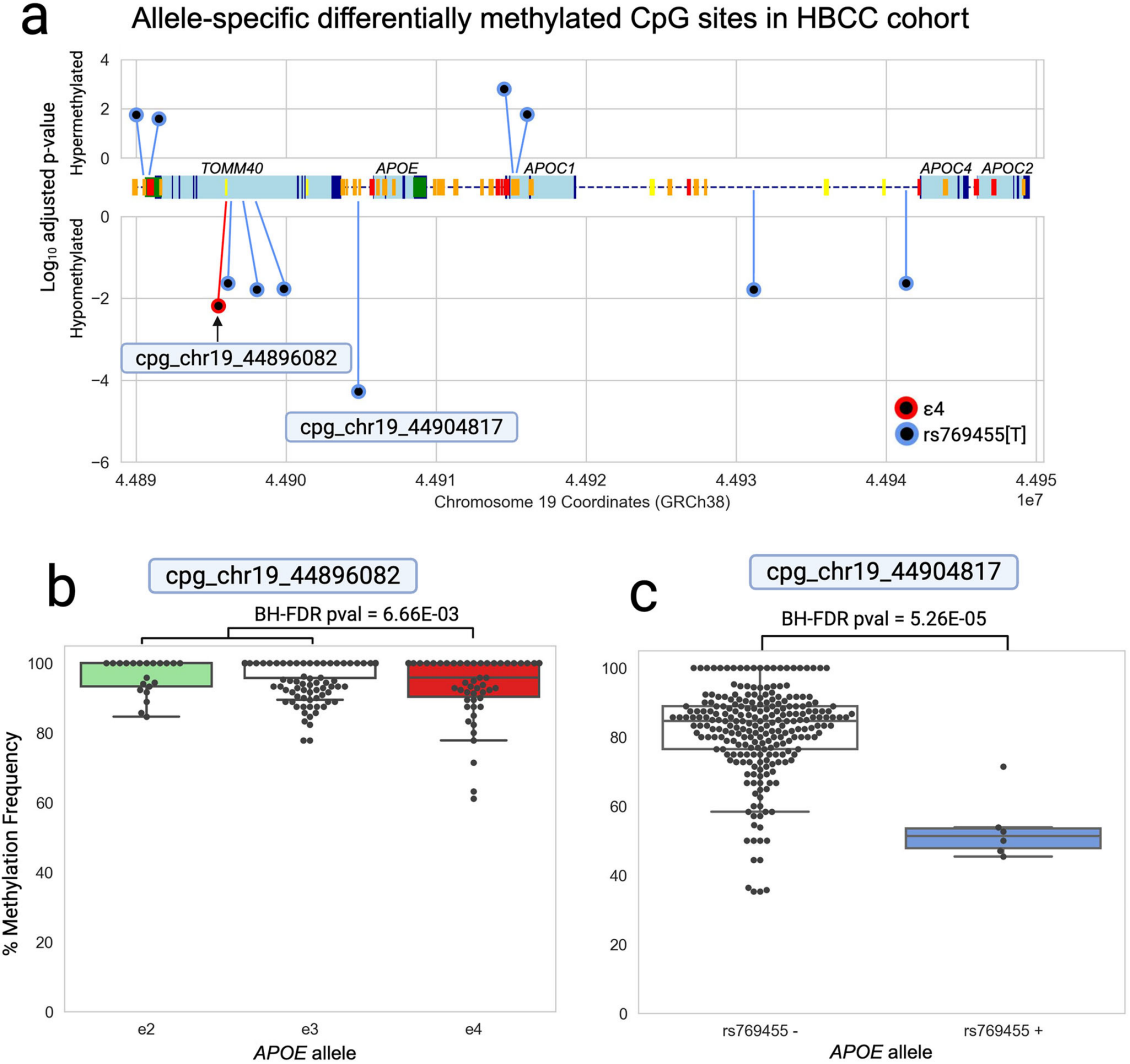
Differentially methylated CpG sites in the HBCC APOE cluster associated with *APOE* ε4 and rs769455[T] in HBCC brain samples. **a** Association of *APOE* ε4 allele (red) and the rs769455[T] variant (blue) with methylation at DMCs within the *APOE* gene cluster. Each dot represents a CpG site that was deemed significantly differentially methylated (BH-FDR-corrected *p* value < 0.05). The y-axis measures the log_10_ adjusted *p* value, with positive numbers indicating hypermethylation associated with the allele or variant and negative numbers indicating hypomethylation associated with the allele or variant (in comparison to sample haplotypes without the allele/variant). The labeled CpG sites are graphed in 4b and c. A schematic of the *APOE* cluster region is shown in the middle x-axis and the bottom x-axis depicts chromosome 19 genome coordinates (reference GRCh38). **b** Box-and-whisker plot showing methylation frequencies associated with the *APOE* ε4 allele at CpG site cpg_chr19_44896082, stratified by *APOE* allele status. **c** Box-and-whisker plot showing methylation frequencies associated with the rs769455[T] variant at CpG site cpg_chr19_44904817.

Bolded CpG sites are visualized in Figs. 3b, c and 4b, c.

*SE* standard error.

The most significant methylation difference was observed at DMC cpg_chr19_44917997 in *APOC1* intron 3, where *ε*4 alleles were associated with decreased methylation compared to non-*ε*4 alleles (BH-FDR-corrected *p* value = 4.90 × 10^−32^, beta = –29.74, SE = 2.13, *P* = 3.15 × 10^–35^; Fig. 3c, Table 2). This CpG site is immediately adjacent to *APOC1* intronic variant rs12721046(G > A) which destroys a CpG site and is in linkage disequilibrium with *APOE ε*4 (LD r^2^ = 0.6, LD D’ = 0.83)^39^. This variant is associated with increased AD risk on the background of the *APOE ε*4 allele in non-Hispanic white populations^40–43^. An allele-specific linear regression analysis (described in the “Methods” section) was performed at the rs12721046(G > A) site and yielded a BH-FDR-corrected *p* value of 1.22 × 10^−27^ when *APOE ε*4 was included as a covariate, whereas the *APOE ε*4 allele yielded an insignificant BH-FDR-corrected *p* value of 0.98 when *APOC1* rs12721046[A] was included as a covariate (Supplementary Fig. S4). This pattern reflects the strong linkage disequilibrium between *APOE ε*4 and rs12721046[A] in European populations, and suggests that the methylation difference at cpg_chr19_44917997 is more tightly correlated with *APOC1* rs12721046[A] variant than the *APOE ε*4 allele.

In the HBCC cohort (*n* = 131; AFR and AFR-admixed ancestry), 11 CpG sites exhibited significant allele-specific methylation differences after BH-FDR correction (FDR < 0.05). One site showed *APOE* ε4-associated differential methylation (cpg_chr19_44896082, Fig. 4b), and the remaining 10 were associated with the rs769455[T] variant. All 11 sites were novel and none were detected as significantly differentially methylated in the NABEC cohort. These DMCs were distributed across diverse genomic features: three were located in promoter or CpG island regions, four in exons and exon transcripts, six in introns, and four in intergenic regions (Table 2). The strongest association was observed at cpg_chr19_44904817, where haplotypes carrying the rs769455[T] variant were hypomethylated relative to those carrying the reference (BH-FDR-corrected *P* = 5.26 × 10^−5^, beta = –30.4, SE = 5.49, *P* = 1.03 × 10^–7^; Fig. 4c, Table 2).

To evaluate the impact of haplotype phasing on DMC detection, we applied the same OLS linear regression framework used in the allele-specific analysis to unphased, allele-averaged methylation counts in the NABEC and HBCC cohorts. For each CpG site, we tested the association between ε2 and ε4 allele dosage and methylation levels. We refer to this approach as ‘allele-averaged’ since methylation levels are averaged across both haplotypes. For example, a CpG site that was 0% methylated on haplotype 1 and 100% methylated on haplotype 2 would be interpreted as 50% methylated in the allele-averaged analysis (Fig. 1b).

In the NABEC samples, the allele-averaged approach identified only three of the DMCs detected in the allele-specific analysis (cpg_chr19_44914329 (ε2), cpg_chr19_44917997, cpg_chr19_44921919), and both were less significant in the allele-averaged analysis than in the allele-specific analysis (Table 2; Supplementary Table S4). None of the allele-specific HBCC methylation differences were found to be significant in the allele-averaged methylation analysis (Supplementary Table S4), and no *APOE* ε2- or ε4-associated DMCs were shared between the NABEC and HBCC cohorts.

The NABEC DMC cpg_chr19_44914329 provides an example of a more modestly significant DMC that was not detected by the allele-averaged methylation analyses. This CpG site showed significant methylation differences associated with the *APOE* ε4 and *APOE* ε2 alleles (BH-FDR-corrected *p* value = 3.23 × 10^−2^, beta = 8.07, SE = 2.08, *P* = 1.25 × 10^−4^ and BH-FDR-corrected *p* value = 1.46 × 10^-2^, beta = 8.32, SE = 1.96, *P* = 2.81 × 10^–5^; Fig. 3b, Table 2) but did not pass significance thresholds in the allele-averaged methylation analyses based on homozygous *APOE* genotypes, ε2 carrier status, ε4 dosage, or ε4 carrier status (Supplementary Table S4; Supplementary Fig. 5A–F).

NABEC CpG site cpg_chr19_44914329 was also the only DMC in our study that overlapped with findings from previous methylation studies of *APOE* and AD (to our knowledge). Shao et al. (2018) reported differential methylation at this site in blood samples (*P* = 0.019, not significant after multiple testing correction), and Panitch et al. (2024) found that cpg_chr19_44914329 was significantly hypomethylated in *APOE* ε4 carriers compared to non-carriers in control blood samples (*P* = 1.5 × 10^−10^). Both studies used the Illumina Infinium HumanMethylation450 BeadChip array to measure methylation, where cpg_chr19_44914329 corresponds to probe cg2327011 (Supplementary Table S1).

To explore whether *APOE* allele-driven methylation differences affect gene expression, we applied the OLS linear regression analysis to bulk RNA-seq data that was available for all NABEC samples and 86 of the HBCC samples^36^. The expression level of each detectable gene in the *APOE* gene cluster (*TOMM40*, *APOE*, and *APOC1*) was tested for associations with the dosage level of each *APOE* allele (ε2 and ε4), with no significant associations observed (Supplementary Table S5).

An expression quantitative trait methylation (eQTM) analysis was conducted to evaluate whether differential methylation at CpG sites was associated with gene expression levels of the APOE cluster genes. For each CpG–gene pair, linear regression models were fitted using methylation frequency as the predictor and gene expression as the outcome, adjusting for APOE ε2, ε4 and rs769455[T] dosages, demographic covariates, and both genetic and methylation principal components. After multiple-testing correction, none of the NABEC or HBCC CpG sites showed significant associations with expression of *APOE*, *TOMM40*, or *APOC1*, indicating that the genotype-associated methylation differences observed in this region do not produce detectable cis-eQTM effects in these postmortem brain samples. The eQTM results for all CpG sites identified as significantly differentially methylated in the methylation analyses are provided in Supplementary Table S6.

We are also generating a single-nucleus Multiome atlas from the NABEC and HBCC cohorts to study changes in gene expression and chromatin accessibility across aging (under review). The nuclei used for this analysis were isolated from tissue adjacent to the regions used for long-read methylation sequencing. Total cell counts and estimated cell-type fractions were calculated from the processed single-nucleus dataset and used to explore whether variation in cellular composition may influence methylation levels. The estimated cell-type composition of the NABEC samples with snMultiome data (*N* = 168) was 7.31% astrocytes, 23.22% excitatory neurons, 13.32% inhibitory neurons, 6.02% microglia, 5.74% oligodendrocyte precursor cells (OPCs), 43.29% mature oligodendrocytes, and 0.95% vascular cells. The processed HBCC samples (*N* = 127) were 12.71% astrocytes, 28.18% excitatory neurons, 14.88% inhibitory neurons, 5.23% microglia, 6.50% OPCs, 31.09% mature oligodendrocytes, and 1.40% vascular cells (Supplementary Table S7). The sample size (N) reflects the number of individuals with DNA methylation data in the analyzed region, complete metadata, and corresponding single-nucleus Multiome data.

To identify CpG sites whose methylation levels were associated with inferred cell-type composition, we fit an OLS linear regression model for each CpG site in the *APOE* gene cluster region. Methylation proportion was used as the outcome, cell-type fractions as predictors, and *APOE* ε2 and ε4 allele dosage, demographic covariates, and genetic principal components were included as covariates. No significant cell-type–associated methylation effects were detected in the NABEC or HBCC cohorts, suggesting that bulk cell-type composition does not substantially account for the *APOE*-related methylation differences observed in this study.

## Discussion

This study represents the most comprehensive analysis to date of allele specific DNA methylation at the *APOE* locus in the human brain using long-read sequencing. We leveraged publicly available data from the NIH CARD Long-Read Sequencing Initiative to analyze 332 postmortem brain samples from individuals of EUR and AFR ancestry, generating phased methylomes that captured a level of epigenetic resolution previously inaccessible with array and bisulfite based techniques. ONT LRS detected ~33-fold more CpG sites across the *APOE* gene cluster than the Illumina Infinium HumanMethylation450 BeadChip array, extending coverage beyond promoter and exonic regions into intronic, intergenic, and enhancer-associated elements. This expanded view of the brain methylome facilitated the discovery of 18 DMCs that showed differential methylation between haplotypes carrying the *APOE* ε2 and ε4 alleles and the rs769455[T] variant. These DMCs were enriched in regions with known regulatory potential, including promoters, CpG islands, enhancers, introns, and SINE elements, offering a more detailed map of epigenetic variation within the *APOE* locus that may contribute to AD risk.

One of the few CpG sites within the *APOE* locus that overlapped with an Illumina Infinium HumanMethylation450 BeadChip array probe was cpg_chr19_44914329, which is also the only CpG detected in our study that we found to be reported in previous methylation analyses. Panitch et al. (2024) found that this site was hypomethylated in peripheral blood samples from *APOE* ε4 carriers compared to non-carriers. In contrast, our brain-based analysis revealed that cpg_chr19_44914329 was hypermethylated on ε4 alleles relative to non-ε4 alleles. This discrepancy may reflect well-documented tissue-specific methylation differences across the genome^44–46^. It may also indicate that brain and blood respond differently to *APOE* ε4 protein levels due to unique physiological demands or compensatory mechanisms^47^. It is important to consider that the pleiotropic and tissue-specific nature of genetic variants may lead to distinct and sometimes divergent epigenetic signatures in different biological systems.

Previous studies investigating allele-specific methylation at the *APOE* locus have primarily focused on the ε4 allele due to its strong association with AD. The ε2 allele is less studied, mainly because its lower allele frequency leads to reduced statistical power in most cohorts. Using phased data and an allele-specific analytical framework, we analyzed 73 ε2 alleles (48 from NABEC and 25 from HBCC) and identified three DMCs in the NABEC cohort with differential methylation on ε2-carrying haplotypes, two of which were located in putative regulatory elements. Although we cannot determine whether these methylation patterns mediate the protective effects of ε2, they provide a framework for future studies to test whether allele-associated epigenetic variation influences *APOE* function or downstream AD-related pathways. These findings provide some of the first evidence of ε2-specific methylation patterns in the human brain and offer new insights into the epigenetic mechanisms that may underlie the allele’s protective role in AD.

Despite these advances, several limitations should be considered. The NABEC and HBCC samples were sequenced using different ONT chemistries (R9 and R10, respectively), which may introduce technical variation in variant and methylation calling. Subtle methylation differences detected in allele-specific analyses are particularly susceptible to chemistry-related biases, which complicates direct comparisons across cohorts^35^. Our interpretation assumes that the effects of chemistry are modest relative to the allele-associated methylation differences observed. While this appears reasonable given the strength of the allele-specific signals, it remains an important caveat when comparing cohorts sequenced with different sequencing chemistries^48^.

Additionally, sample size remains a limiting factor, particularly in the African ancestry cohort, where the number of individuals carrying the ε2 allele or rs769455[T] variant was relatively small. This limited our power to detect allele-specific methylation differences and prevented genotype-based modeling for certain variants. Expanding representation of ancestrally different populations will be critical to fully capture the regulatory landscape of *APOE* and its functional variants.

There were also no shared DMCs detected in the NABEC and HBCC cohorts. This absence may be a reflection of meaningful biological and statistical differences between the cohorts. HBCC included fewer samples resulting in reduced power, and several NABEC DMCs showed trends toward significance in HBCC but did not surpass FDR thresholds. Ancestry-related methylation differences may also contribute, as multiple studies have shown that genetic ancestry strongly influences both global and locus-specific methylation patterns^49,50^. Because most prior methylation studies of the human frontal cortex have focused on individuals of European ancestry, our analysis of African-ancestry HBCC samples likely uncovered cohort-specific or ancestry-associated DMCs not observed in NABEC or reported in previous literature.

We evaluated potential gene expression associations of differentially methylated CpG sites using bulk RNA-seq data from the NABEC and HBCC cohorts. The RNA-seq data was generated using SRS, which limits resolution for full-length transcripts and reduces the ability to quantify allele-specific expression, particularly in polymorphic regions such as the *APOE* locus. Although no significant eQTLs or eQTMs were identified, this may reflect the limited resolution of SRS data rather than a true absence of regulatory effects. Future studies using long-read RNA sequencing will be critical for resolving isoform diversity and quantifying allele-specific transcript expression, enabling a more accurate assessment of the functional consequences of methylation variation.

The absence of significant associations between CpG methylation and estimated cell-type proportions suggests limited evidence for strong cell-type–driven methylation effects in this region. However, interpretation is constrained by an important limitation: the single-nucleus transcriptomic datasets used to estimate cell-type proportions were generated from tissue *adjacent to*, but not identical to, the cortical punches used for long-read methylation profiling. Even small spatial differences in cellular composition may reduce sensitivity to detect true cell-type–dependent methylation patterns^51,52^.

Emerging deconvolution approaches that operate directly on phased long-read methylation data, such as CellFie-ISH^53^, offer a promising solution but currently require a high-quality reference methylation atlas for human brain cell types. Such an atlas does not yet exist. Ongoing NIH–CARD efforts to generate phased long-read methylomes across major neuronal and glial subtypes will eventually enable accurate deconvolution from the same bulk DNA samples analyzed here, reducing reliance on adjacent-tissue proxies and improving power to identify cell-type–specific *APOE*-allele-associated methylation differences.

All samples in this study came from neurologically healthy control brains. The lack of observed expression changes may therefore reflect the absence of disease-related transcriptional dysregulation. Investigating these methylation patterns, and their regulatory impacts, in Alzheimer’s disease and related dementia (ADRD) cohorts will be essential for understanding their potential contribution to disease risk and progression.

Looking ahead, this work provides a strong foundation for future functional studies to test whether *APOE* allele-specific methylation differences influence gene regulation or contribute to disease-related pathways. Such studies could include allele-specific reporter assays to directly test the regulatory activity of methylated versus unmethylated *APOE*-linked CpGs, CRISPR-based epigenome editing to perturb methylation states at rs769455[T]-associated CpGs and measure downstream transcriptional or lipid-processing effects, and chromatin conformation assays (e.g., Capture-C or HiChIP) to determine whether allele-specific methylation alters enhancer–promoter interactions across the *APOE* locus. These approaches would help clarify whether the methylation differences identified here have causal roles in *APOE* regulation or AD-related molecular pathways.

Thousands of additional brain samples, including ones from individuals affected by ADRDs, are currently being sequenced with improved R10 chemistry as part of NIH’s CARD Long-Read Sequencing Initiative. Expanding these datasets will further refine the resolution of *APOE* haplotype diversity and methylation architecture. In parallel, ongoing single-nucleus sequencing efforts and forthcoming long-read RNA-seq profiling of the NABEC and HBCC cohorts will provide cell-type–resolved and isoform-level expression data, offering new opportunities to link allele-specific methylation to gene regulatory outcomes.

In summary, our study highlights the advantages of long-read sequencing for resolving the complex haplotype structure and epigenetic landscape of the *APOE* locus in human brain tissue. These findings offer new insights into how *APOE* genetic variation shapes methylation patterns and establish a foundation for dissecting the molecular mechanisms that link *APOE* haplotypes to AD-related cellular dysfunction.

## Methods

### Cohort information

Post-mortem tissue research is classified as non-human subjects research by the National Institutes of Health and it does not fall under IRB jurisdiction. We utilized existing ONT WGS data from the North American Brain Expression Consortium (NABEC) (dbGaP accession phs001300.v6.p1) comprising 206 prefrontal brain tissue samples from individuals of European ancestry obtained from the following brain banks and institutions: the University of Maryland and Sun Health Brain Banks, Kentucky Alzheimer’s Disease Research Center (ADRC), the Baltimore Longitudinal Study on Aging at the Johns Hopkins School of Medicine, the National Institute of Child Health and Human Development (NICHD) Brain and Tissue Bank for Developmental Disorders at the University of Maryland, the Banner Sun Health Research Institute Brain and Body Donation Program, the National Institute on Aging, the Arizona Department of Health Services, the Arizona Biomedical Research Commission, the Michael J. Fox Foundation for Parkinson’s Research, and the University of Kentucky Alzheimer’s Disease Center Brain Bank. Donor consent and ethical oversight were managed by the source brain banks in accordance with their institutional protocols. All NABEC samples used in this study carried Consent Code 1 (General Research Use, GRU), indicating that broad consent for research use had been obtained from donors or their legally authorized representatives. The African admixed sample set (*n* = 155) was provided by the Human Brain Collection Core (HBCC) at the National Institute on Mental Health (ZIC MH002903). All HBCC samples were collected with broad research permissions from the next-of-kin, with procedures approved by the HBCC Oversight Committee and the NIH Department of Bioethics. All research involving human post-mortem brain tissue samples and associated data was performed in accordance with the Declaration of Helsinki. Sample ancestries were determined genetically using the GenoTools package^54^. All samples were derived from individuals with no known neurological diseases or clinical history of cognitive impairment. Four of the NABEC samples and ten of the HBCC samples were excluded from the study due to receiving non-European (NABEC) or non-African or African admixed (HBCC) genetic ancestry estimations by the GenoTools package^54^, and one sample from each cohort was excluded due to low read coverage across the *APOE* gene cluster region. Additional information about cohort demographics and sequencing statistics can be found in Supplementary Tables S2 and S3.

### Data sources and processing

We accessed preprocessed, aligned BAM files previously generated and publicly released by Billingsley et al. (2024). Detailed protocols for DNA extraction, library preparation, and sequencing are available on the protocols.io platform^55,56^. Briefly, DNA was extracted from approximately 40 mg of homogenized frozen frontal cortex tissue and sheared to a target fragment size of 30 kb. NABEC samples were prepared using the ONT SQK-LSK110 kit and sequenced on R9.4.1 flow cells, while HBCC samples were prepared using the SQK-LSK114 kit and sequenced on R10.4.1 flow cells. All sequencing was performed on PromethION flow cells (FLO-PRO002), with 2–3 rounds of loading to achieve minimum yield thresholds. The NABEC samples were sequenced using MinKNOW versions 22.03.2 through 22.10.7 and basecalled with Guppy v6.1.2, while the HBCC samples were sequenced with MinKNOW v22.10.7 and basecalled using Guppy v6.3.8. Read alignment to the GRCh38 reference genome was performed using minimap2 (v2.23-r1111)^57^.

### Haplotype phasing and methylation calling

Reads were aligned to the GRCh38 reference genome using minimap2^57^ followed by haplotype phasing and downstream methylation analysis. Reads from chromosome 19 were subset from each sample, and phasing was conducted using PEPPER-Margin-DeepVariant (ONT_R9 model) for NABEC samples and DeepVariant (ONT_R10.4 model) for HBCC samples^58–60^. The --dv_sort_by_haplotypes parameter was applied to produce haplotype-resolved BAMs. DeepVariant output gVCFs were used to determine each sample’s *APOE* haplotypes. Original unphased BAMs were retained to compare phased versus unphased methylation profiles.

Methylation calling was carried out on both phased and unphased BAM files using modkit pileup (https://github.com/nanoporetech/modkit), with the --combine-strands and --ignore h options enabled to extract CpG-level methylation frequencies. This pipeline enabled us to quantify allele-specific methylation differences across the *APOE* locus. A schematic overview of the LRS allele-specific and SRS allele-averaged methylation analysis workflows is presented in (Fig. 1b).

### Selection and classification of *APOE* alleles

We selected alleles and variants for this study that were: (i) located within the *APOE* gene cluster; (ii) had a minor allele frequency (MAF) > 0.01 in individuals of European (EUR) and/or African and African admixed (AFR) ancestry; (iii) were classified as protective or pathogenic (CADD PHRED > 20); and (iv) had previously reported being significantly associated with AD risk. The following alleles and variant met these criteria: *APOE* ε2 (rs7412(C > T)), ε4 (rs429358(T > C)), and rs769455(C > T).

### Dendrogram generation

Dendrograms were generated using the pipeline described by Chang et al (2021) (https://bioinformaticsworkbook.org/phylogenetics/FastTree.html#gsc.tab=0) with several adaptations. Briefly, *bedtools*^61^ was used to extract reads in the *APOE* gene region (chr19:44889556–44953378; hg38) from both phased and unphased BAM files in the NABEC and HBCC cohorts. *Samtools*^62^ was then used to split the phased reads by haplotype using the HP tag and each haplotype was treated as an individual sample (i.e. HBCC_81925_FTX becomes HBCC_81925_FTX_H1 and HBCC_81925_FTX_H2). *Bedtools genomecov* was used to filter and include haplotypes with ≥10× coverage for the phased dataset and ≥20× coverage for the unphased dataset.

Each BAM file was then converted into its consensus FASTA sequences using *samtools consensus*. A NABEC sample that had been sequenced with both R9 and R10 chemistries was included to assess whether dendrogram separations were driven by chemistry differences. A FASTA file of the ancestral chimpanzee *APOE* sequence was also included to root the tree^38^. All FASTA sequences were concatenated and aligned using the *Mafft*^63^ package with default parameters, and phylogenetic trees were constructed using the *FastTree* package^64^. The resulting Newick files were visualized using *FigTree* (https://github.com/rambaut/figtree/) with aligned tip labels, ordered nodes, and coloring based on allele or variant type.

### OLS linear regression methylation analyses

Allele-specific DMCs in the *APOE* cluster region were identified using an ordinary least squares linear (OLS) regression model with the presence or absence of the allele or variant as the independent variable and the modkit-determined CpG methylation frequencies as the dependent variable. Covariates included age, sex, post-mortem interval (PMI), groups within the datasets representing brain bank sources (‘SH’, “KEN”, and “UMARY” for NABEC), and genetic and methylation principal components (PCs). Genetic PCs were generated using PLINK (v1.9), with the exclusion of variants with a minor allele frequency <0.05, genotyping rate <0.95, and Hardy-Weinberg equilibrium *p* value > 0.0001. Genome-wide CpG methylation PCs were also generated using PLINK. The number of PCs to include in the analysis was determined using scree plots (first 14 genetic PCs and first 5 methylation PCs were included for both NABEC and HBCC cohorts). Methylation PCs for haplotype 1 and 2 were highly correlated, so only haplotype 1 PCs were used in the analysis. A parallel OLS linear regression analysis was performed using unphased methylation levels to assess which CpG sites would be detectable without haplotype phasing. Genetic principal components were reused from the allele-specific analysis, while methylation principal components were recalculated from genome-wide unphased CpG methylation frequencies. CpG sites with Benjamini–Hochberg false discovery rate (BH-FDR)–adjusted *p* values < 0.05 were considered significant.

Prior to the regression analysis, CpG sites were filtered to ensure data quality. CpGs were retained if they had a minimum coverage of ≥5× for the allele-specific (phased) analysis and ≥10× for the allele-averaged (unphased) analysis.

More details about the files, file formats, and scripts used in the OLS regression model are located on the NIH-CARD Github page (https://github.com/NIH-CARD/CARDlongread_data_standardization) and described in the Long-read sequencing data standardization standard operating procedure. This protocol was originally designed to perform QTL assessments of SNVs and structural variants with modkit-determined CpG methylation frequencies that have been averaged over specific regions of interest; it was adapted to test the associations of the the two *APOE* alleles (ε2, ε4) and the rs769455[T] SNV with the methylation frequencies of each CpG site in the *APOE* gene cluster region.

### Expression linear regression analysis

Bulk RNA-seq data for all of the NABEC samples^65^ and 86 of the HBCC samples (https://nda.nih.gov/edit_collection.html?id=3151) previously processed by Billingsley et al (2024) using Salmon^66^ with the filtered Gencode v43 human transcriptome index. In the NABEC samples, 26,778 genes were considered to be well-detected (missingness under 0.25) including the *TOMM40*, *APOE* and *APOC1* genes. In the 86 HBCC samples, 26,679 genes were considered to be well-detected, including the *TOMM40, APOE, APOC1* genes. The *APOC4-APOC2* gene was detected in the HBCC data but not the NABEC. Expression data was quantile transformed and scaled from 0 to 1. *APOE* allele-related expression differences in the detected *APOE* cluster genes were tested for using an OLS regression model with the presence or absence of the allele as the independent variable and the standardized gene expression data as the dependent variable. Covariates included age, sex, post-mortem interval (PMI), brain bank sources (‘SH’, “KEN”, and “UMARY” for NABEC), and genetic and expression principal components. Genetic PCs were generated using PLINK (v1.9), with the exclusion of variants with a minor allele frequency <0.05, genotyping rate <0.95, and Hardy-Weinberg equilibrium *p* value > 0.0001. Gene expression PCs were calculated using the sklearn.decomposition PCA module. The number of PCs to include in the analysis was determined using scree plots (the first 14 genetic PCs and first five expression PCs were included for both NABEC and HBCC cohorts). Gene expression differences with a BH-FDR corrected *p* value < 0.05 were considered significant.

### eQTM linear regression analysis

To examine whether APOE-associated differentially methylated CpG sites were correlated with local gene expression, we performed an expression quantitative trait methylation (eQTM) analysis using an APOE genotype-adjusted linear regression model. For each CpG site with available methylation frequency estimates and for each target gene, we fit an OLS model in which gene expression was regressed on CpG methylation frequency while adjusting for *APOE* and ε2 and ε4 dosages, age, sex, postmortem interval (PMI), diagnostic group, and the first five genetic principal components (PCs). Models with fewer than 60 complete observations in the NABEC cohort and 30 complete observations in the HBCC were excluded. Significant eQTMs were defined as BH-FDR-adjusted *p* < 0.05.

### Cell-type proportion data from single cell study

To identify CpG sites whose methylation levels were associated with inferred cell-type composition, we fit an OLS linear regression model for each CpG, using methylation proportion as the outcome and cell-type fractions as predictors, while adjusting for age, sex, PMI, *APOE* ε2 and ε4 allele dosage, and the first 5 genetic principal components. Associations were tested per CpG–cell-type pair, and BH-FDR correction was applied separately for each cell type. CpG sites exhibiting significant cell-type–specific methylation differences after BH-FDR correction (FDR < 0.05) were considered significant.

### Cell-type proportions from single nuclei expression dataset

Single nuclei multiome data was collected using the 10x Gemomics Multiome ATAC + expression kit (10x Genomics, 1000283). Tissue was first homogenized and single nuclei were isolated. Libraries were completed as directed in the user guide (10x Genomics, CG000338 Rev. E) and sequenced on an Illumina a NovaSeq6000 S4 (PE150). Resulting FASTQ files were aligned to the transcriptome (reference vGRCh38-2024-A) using Cell Ranger ARC (v2.0.2). The resulting gene by cell matrix was filtered and processed with the scMAVERICS pipeline (github.com/NIH-CARD/scMAVERICS/tree/PFC, 10.5281/zenodo.18135365). Cell types were defined by expression of canonical marker genes. The cell-type proportions used here were calculated by sample, retaining only the samples that had >1000 nuclei after filtering. All processed data is deposited at Zenodo under accession number 17066184.

### Reference methylation data from the ROSMAP study

DNA methylation data shown in Fig. 2a and Supplementary Fig. 1 were derived from the Religious Orders Study and the Rush Memory and Aging Project (ROSMAP) cohorts^37,67^. Data were provided by the Rush Alzheimer’s Disease Center, Rush University Medical Center, Chicago. The results published here are in whole or in part based on data obtained from The AD Knowledge Portal (10.7303/9618239). We analyzed data from the previously produced Illumina HumanMethylation450 BeadChip array, generated from the dorsolateral prefrontal cortex tissue of 697 participants, including 417 individuals with AD and 280 cognitively normal controls. ROSMAP data and biomaterials were collected from several National Institute on Aging (NIA) and National Alzheimer’s Coordinating Center. Quality control checks and preparation of the gene expression data was provided by the National Institute on Aging Alzheimer’s Disease Data Storage Site at the University of Pennsylvania.

## Supplementary information

Supplementary Information

## Data Availability

Access requests for these controlled datasets are available through dbGaP under accessions phs001300.v5.p1 and phs000979.v4.p2 and the data is available at https://explore.anvilproject.org/datasets or https://duos.org/datalibrary/anvil. The code used to process and analyze the data for this study is publicly available at https://github.com/NIH-CARD/APOE_CpG_methylation. This repository includes scripts designed to conduct an allele-specific, CpG-site specific beta-binomial regression analysis given a modkit bed file for a specific region and sample haplotype designations. The scripts used to generate all main and supplementary figures are also included. Additional details about the files and file formats and the scripts used to generate the PC values are located on the NIH-CARD Github page (https://github.com/NIH-CARD/CARDlongread_data_standardization).
