# Supplementary material for: Haplotype-resolved DNA methylation at the APOE locus identifies allele-specific epigenetic signatures relevant to Alzheimer’s disease risk
Source: NPJ Dement. 2026 Jun 19;2(1):45. doi: 10.1038/s44400-026-00094-8 (PMC13282175; doi:10.1038/s44400-026-00094-8)
Supplement: Supplementary file 1 — Supplementary Information [file 44400_2026_94_MOESM1_ESM.pdf]

Supplementary Figures:

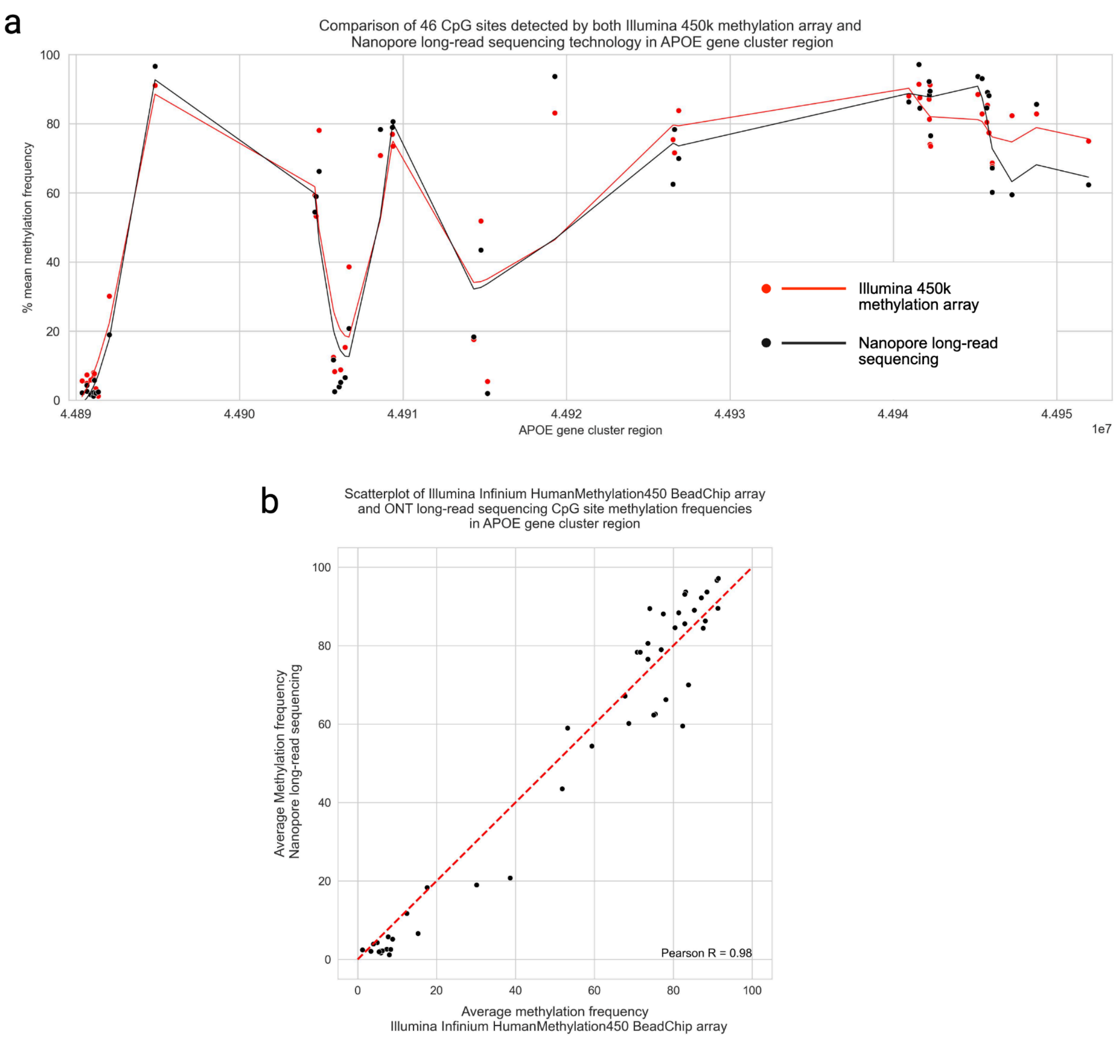

Supplementary Figure 1. Comparison of the 46 CpG sites detected by both platforms. a) Mean methylation frequencies of the 46 CpG sites detected in two separate EUR-ancestry brain tissue cohorts sequenced by the Illumina Infinium HumanMethylation450 BeadChip array (red) and the ONT long-read sequencing technology (black) within the *APOE* cluster region (*TOMM40, APOE, APOC1,* and *APOC4-APOC2* genes; hg38 coordinates chr19:44889556-44953378). **b)** Scatter plot of the Illumina Infinium HumanMethylation450 BeadChip array and the long-read sequenced CpG site methylation frequencies in the *APOE* cluster region. Pearson R = 0.98, P-value = 3.506×10^{-33}.

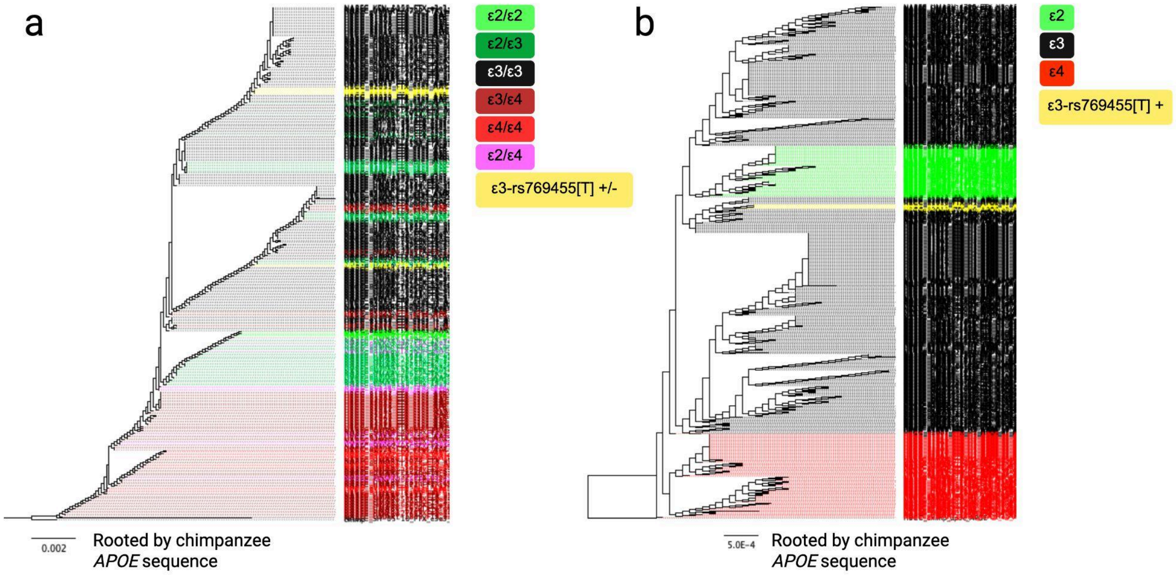

Supplementary Figure 2. Dendrogram depictions of *APOE* allele separation at the allele and genotype levels. a) Dendrograms of the *APOE* gene region (chr19:44905791-44909393, hg38) generated from 332 unphased genotypes from the NABEC and HBCC cohorts. Branches are colored by *APOE* genotype. Only basecalls with >20× coverage were included. **b)** Dendrogram of the *APOE* region generated from 664 phased haplotypes from the NABEC and HBCC cohorts. Branches are colored by allele type: *APOE* ε2 in green, *APOE* ε4 in red, and rs769455[T] in yellow. Only positions with > 10× coverage were included. In both panels, each branch represents the aligned consensus FASTA sequence for an individual *APOE* genotype (a) or haplotype (b). Dendrograms were rooted using the ancestral chimpanzee *APOE* sequence. See Methods for full details on dendrogram generation.

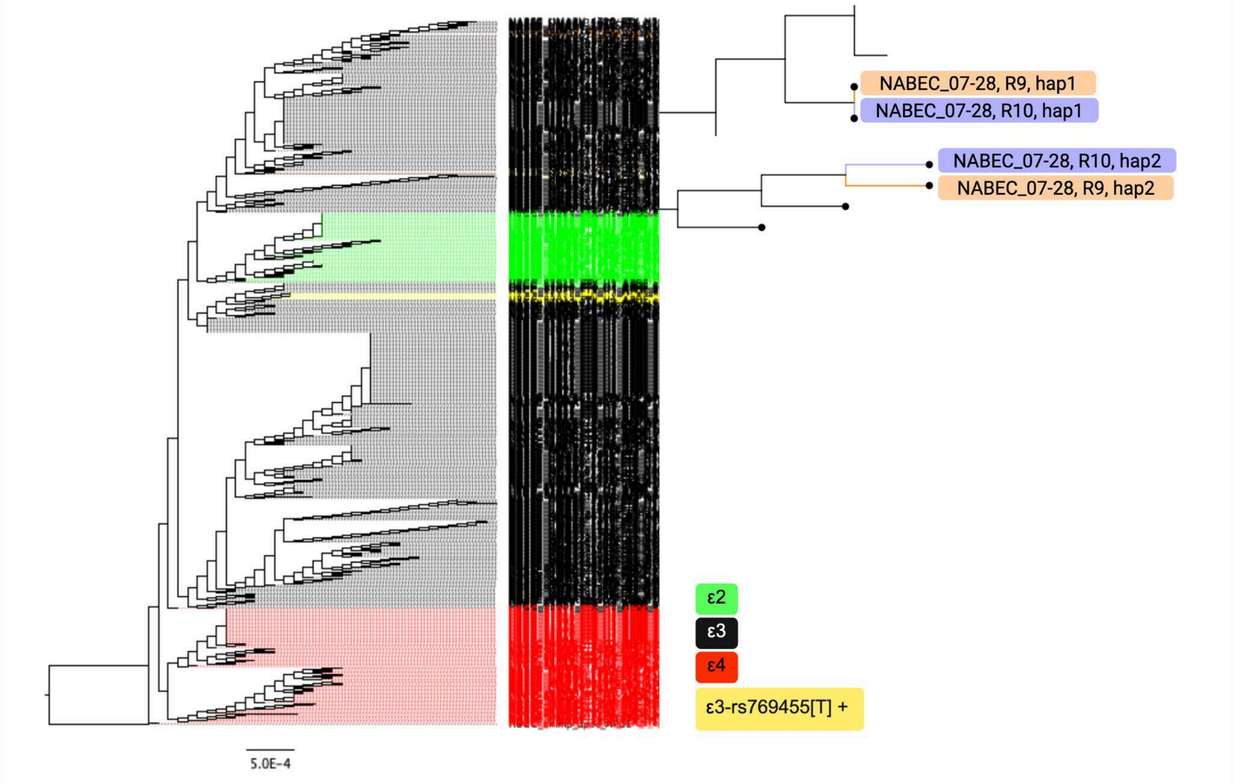

Supplementary Figure 3. Benchmarking the effect of Nanopore flow cell chemistry on phased allele separation in a dendrogram. A copy of the dendrogram featured in Supplementary Figure 2 has been expanded to show one NABEC sample (NABEC_07-28) that was sequenced with both R9 (orange) and R10 (purple) chemistries. The branches containing each sample haplotype have been magnified on the right side of the dendrogram.

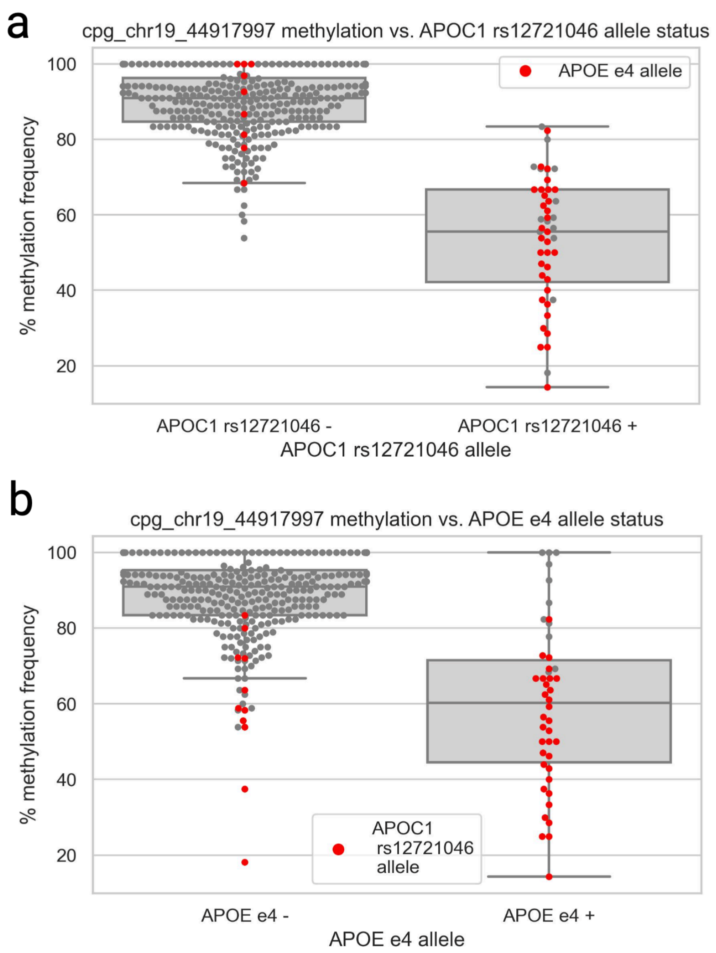

Supplementary Figure 4. Assessing *APOE* e4 and *APOC1* rs12721046[A] allele effects on methylation frequency differences at NABEC CpG site cpg_chr19_44917997. a) A box-and-whisker plot showing the methylation frequency difference at NABEC CpG site cpg_chr19_44917997 when stratified by the *APOC1* rs12721046[A] allele (with the *APOE* ε4 alleles highlighted in red and included as a covariate in the linear regression analysis). **b)** A box-and-whisker plot showing the methylation frequency difference at NABEC CpG site cpg_chr19_44917997 when stratified by the *APOE* ε4 allele (with the *APOC1* rs12721046[A] alleles highlighted in red and included as covariate in linear regression analysis). The dots on the left half of the box plots depict sample haplotypes that do not have the allele of interest (denoted by "-") and the right half show samples that do have the allele of interest (denoted by "+").

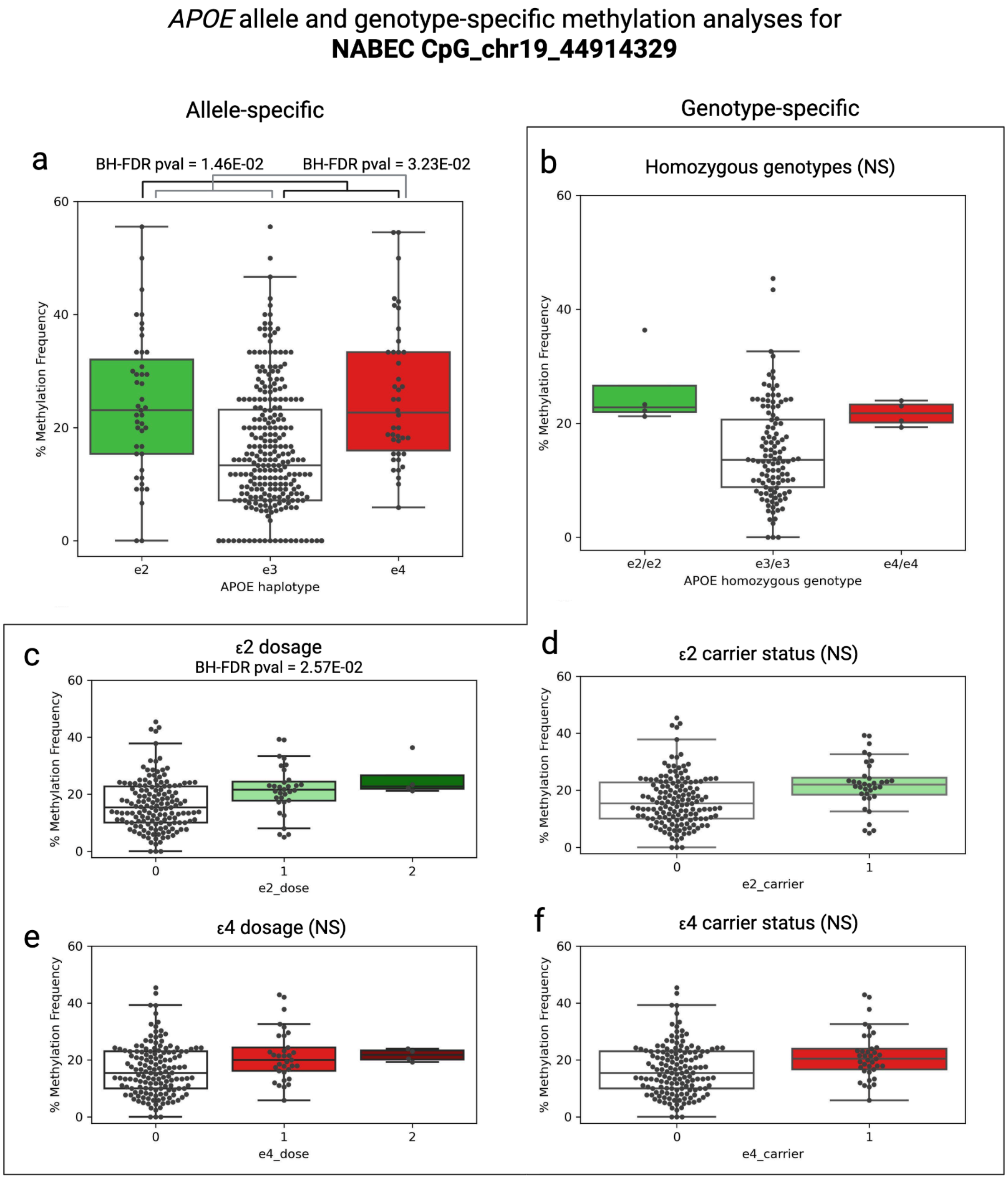

Supplementary Figure 5. Box-and-whisker plots of the ε4 APOE allele-specific methylation frequencies for NABEC CpG site cpg_chr19_44914329 as determined by allele-specific and genotype-specific methylation analyses. **a)** Allele-specific methylation analysis. **b-f)** Genotype-specific methylation analyses based on homozygous genotypes (b), ε2 dosage and carrier status (c,d) and ε4 dosage and carrier status (e,f). NS = BH-FDR corrected p value was not significant.

Supplementary Tables

Table S1. Summary table of previous studies of APOE methylation in brain and blood tissue samples including the number and type of samples analyzed, methylation sequencing technique used, number of CpG sites identified and where they were located in the genome.

Article	Sample type	Sample groups	Meth. sequencing	# CpG sites	Region
Wang et al. 2008	Prefrontal cortex brain tissue, lymphocytes	Brain: 24 AD, 10 control<br><br>Lymphocytes: 6 AD, 6 control	Bisulfite conversion followed by MALDI-TOF mass spectrometry analysis of base-specific cleavages	123 CpG units	12 potential AD-susceptible loci (including HTATIP, MTHFR, DNMT1, TDAM, SIN3A, NCSTN, BACE1, APP, PSEN1, APG1B, and APOE genes)
Yu et al. 2013	Multiple (cerebellum, hippocampus, frontal and temporal lobe, whole-blood lymphocytes)	9 AD<br>6 age-matched controls	Bisulfite pyrosequencing	75 CpG sites	APOE exon 4 CGI
Foraker et al. 2015	Brain tissue (frontal and temporal lobes, hippocampus, cerebellum)	15 AD<br>10 controls	Bisulfite pyrosequencing	76 CpG sites	APOE exon 4 CGI
Ma et al. 2015	Blood T lymphocytes (GOLDN), various cell types (ENCODE)	993 participants from Genetics of Lipid Lowering Drugs and Diet Network (GOLDN) and ENCODE	Illumina Infinium HumanMethylation 450 BeadChip assay	13 CpG sites	APOE gene
Shao et al. 2018	Human brain - Hippocampus and cerbellum / Peripheral blood	Brain: 12 / Peripheral blood: 67	Illumina Infinium HumanMethylation 450 BeadChip Kit	54 CpG sites	TOMM40, APOE, APOC1, APOC2
Liu et al. 2018	Peripheral blood leukocytes	289 African americans with hypertension and their siblings	Illumina Infinium HumanMethylation 450 BeadChip assay	48 CpG sites	PVRL2, TOMM40, APOE genes
Karlsson et al. 2018	Peripheral blood leukocytes	447 Swedish twins, mix of dementia and/or AD, CVD, and controls	Illumina HumanMethylation EPIC BeadChip array	13 CpG sites	APOE gene
Mur et al. 2020	Blood samples from individuals between 30-65 years old	5828 blood samples from Generation Scotland (GS) cohort	Illumina HumanMethylation EPIC BeadChip array	13 CpGs	APOE gene (promoter, introns 2 and 3, exons 2, 3, 4)

Walker et al. 2021	Whole blood DNA	All controls<br>2469 e4 carriers<br>1118 e2 carriers<br><br>Discovery sample: 5190<br>Replication sample: 4583	Illumina Infinium MethylationEPIC BeadChip assay	Genome-wide (850,000+ CpG sites)	Genome-wide methylation differences associated with e2 and e4 alleles
Panitch et al. 2024	2021 blood samples<br>697 brain samples	ADNI (blood): 91 AD, 329 MCI, 210 controls<br><br>FHS (blood): 1391 controls<br><br>ROSMAP (brain): 417 AD, 280 controls	HumanMethylation 450 BeadChip array (ROSMAP and FHS)<br><br>Infinium MethylationEPIC BeadChip array (ADNI)	Genome-wide (450,000+ CpG sites)	Genome-wide methylation differences associated with AD status and/or APOE genotype

Table S2: Summary of cohort demographics including the total number samples, number of male and female samples, mean age, age range, ancestry, diagnosis, sample type, sequencing coverage and sequencing N50.

Cohort	N (Total)	N (Males)	N (Females)	Mean Age (SD)	Age Range	Ancestry	Diagnosis / Phenotype	Sample Type	Sequencing Coverage (Mean ± SD)	Sequencing N50 (Mean ± SD)
NABEC	201	129	72	52.37 (27.51)	15-96	European	Control (n=201)	Prefrontal Cortex	12.12 (3.57)	28141.32 (3836.14)
HBCC	131	80	51	45.19 (14.34)	18.1-85.2	African	Control (n=131)	Cortex	10.63 (2.61)	25623.63 (5994.21)

Table S3: Haplotype-specific sequencing statistics for each NABEC and HBCC sample used in the study including the mean and median sequencing lengths, N50, number of reads, and mean coverage.

Cohort	Sample ID	Mean Length	Median Length	N50	Number of Reads	Mean Coverage
NABEC	NABEC_KEN_1066_FTX_H1	14259	8310	26994	97	11.1496
NABEC	NABEC_KEN_1066_FTX_H2	11478	7019	23311	100	10.3034
NABEC	NABEC_KEN_1069_FTX_H1	22747	23531	30325	48	8.47232
NABEC	NABEC_KEN_1069_FTX_H2	23431	25392.5	28571	60	10.5504
NABEC	NABEC_KEN_1070_FTX_H1	12598	8083.5	21564	122	12.9238
NABEC	NABEC_KEN_1070_FTX_H2	12500	8370	23996	128	12.3781
NABEC	NABEC_KEN_1092_FTX_H1	14149	9597	24477	59	6.96524

NABEC	NABEC_KEN_1092_FTX_H2	17829	18462	24390	69	10.6931
NABEC	NABEC_KEN_1106_FTX_H1	20578	20668	29555	67	10.3458
NABEC	NABEC_KEN_1106_FTX_H2	17822	17293	25761	77	10.1748
NABEC	NABEC_KEN_1127_FTX_H1	16780	16602	25748	119	15.0335
NABEC	NABEC_KEN_1127_FTX_H2	14615	11711	23354	114	14.1966
NABEC	NABEC_KEN_1131_FTX_H1	19301	22360	29286	63	9.29096
NABEC	NABEC_KEN_1131_FTX_H2	16394	12155	29722	77	11.3426
NABEC	NABEC_KEN_1132_FTX_H1	20955	21225	31238	50	8.10614
NABEC	NABEC_KEN_1132_FTX_H2	22795	26444	32606	63	10.2366
NABEC	NABEC_KEN_1142_FTX_H1	12046	5979.5	25593	102	9.51892
NABEC	NABEC_KEN_1142_FTX_H2	13033	8755	25609	128	11.1515
NABEC	NABEC_KEN_1153_FTX_H1	22725	28229	32714	85	14.2224
NABEC	NABEC_KEN_1153_FTX_H2	19135	17016	30345	70	11.7129
NABEC	NABEC_KEN_1159_FTX_H1	15939	14027	27518	150	17.4354
NABEC	NABEC_KEN_1159_FTX_H2	14624	10304	29756	150	15.101
NABEC	NABEC_KEN_1161_FTX_H1	18636	13593	34673	106	11.9812
NABEC	NABEC_KEN_1161_FTX_H2	19338	15609	33285	77	8.23956
NABEC	NABEC_KEN_1163_FTX_H1	16370	14061	31201	103	12.5955
NABEC	NABEC_KEN_1163_FTX_H2	15451	12582.5	26879	94	12.1499
NABEC	NABEC_KEN_1177_FTX_H1	16117	12674	27958	66	8.55829
NABEC	NABEC_KEN_1177_FTX_H2	21312	22183	32642	68	10.7868
NABEC	NABEC_KEN_1184_FTX_H1	24427	26045	29087	59	11.7336
NABEC	NABEC_KEN_1184_FTX_H2	25157	25655	32211	73	12.3916
NABEC	NABEC_KEN_1206_FTX_H1	20051	18875.5	30273	74	11.8087
NABEC	NABEC_KEN_1206_FTX_H2	21326	19713.5	30099	76	13.0031
NABEC	NABEC_KEN_1224_FTX_H1	18033	18955	29087	163	19.4692
NABEC	NABEC_KEN_1224_FTX_H2	18185	16726	31473	159	21.7085
NABEC	NABEC_KEN_1229_FTX_H1	18410	14604	33889	105	13.1176
NABEC	NABEC_KEN_1229_FTX_H2	19022	18461	32793	119	15.4097
NABEC	NABEC_KEN_1245_FTX_H1	14724	10584	26639	82	8.88754
NABEC	NABEC_KEN_1245_FTX_H2	15142	11510	28253	87	9.48581
NABEC	NABEC_KEN_5015_FTX_H1	19565	21097	29580	80	10.9964
NABEC	NABEC_KEN_5015_FTX_H2	18975	19318	28066	69	9.90252
NABEC	NABEC_KEN_5034_FTX_H1	18871	18761	22753	88	13.1174
NABEC	NABEC_KEN_5034_FTX_H2	18912	20226	24968	64	10.0064
NABEC	NABEC_KEN_5062_FTX_H1	18230	17292.5	27013	94	14.0911
NABEC	NABEC_KEN_5062_FTX_H2	18482	18984.5	27797	64	10.7181
NABEC	NABEC_KEN_5091_FTX_H1	17683	16782	25326	137	18.1508
NABEC	NABEC_KEN_5091_FTX_H2	18260	15584	29287	162	19.4853

NABEC	NABEC_KEN_5163_FTX_H1	9103	5237	18461	178	14.5848
NABEC	NABEC_KEN_5163_FTX_H2	9368	5225	18424	147	10.8319
NABEC	NABEC_SH_00_34_FTX_H1	20637	22823	30862	111	15.6133
NABEC	NABEC_SH_00_34_FTX_H2	21421	21856	31287	109	16.4393
NABEC	NABEC_SH_00_49_FTX_H1	24203	26924	30939	47	8.85517
NABEC	NABEC_SH_00_49_FTX_H2	23411	27045	31670	57	9.89874
NABEC	NABEC_SH_01_31_FTX_H1	23689	25736	30464	93	15.5645
NABEC	NABEC_SH_01_31_FTX_H2	24095	23722	31466	67	10.258
NABEC	NABEC_SH_01_37_FTX_H1	24782	26014	32845	63	12.2094
NABEC	NABEC_SH_01_37_FTX_H2	18227	16908.5	29319	52	7.34973
NABEC	NABEC_SH_02_08_FTX_H1	21771	23259	29798	75	12.6086
NABEC	NABEC_SH_02_08_FTX_H2	22736	25208.5	30681	72	12.0707
NABEC	NABEC_SH_03_15_FTX_H1	24497	23340.5	27598	52	8.46014
NABEC	NABEC_SH_03_15_FTX_H2	19311	18891	25351	61	8.2594
NABEC	NABEC_SH_03_17_FTX_H1	20704	23066	32064	71	10.8094
NABEC	NABEC_SH_03_17_FTX_H2	22193	24053.5	31339	68	10.9975
NABEC	NABEC_SH_04_05_FTX_H1	19798	20532	29485	135	20.5187
NABEC	NABEC_SH_04_05_FTX_H2	22300	22622.5	29413	134	21.1713
NABEC	NABEC_SH_04_08_FTX_H1	19311	16331.5	30607	78	13.1577
NABEC	NABEC_SH_04_08_FTX_H2	20670	20687	32593	101	13.485
NABEC	NABEC_SH_04_19_FTX_H1	20898	21350	30563	124	18.1338
NABEC	NABEC_SH_04_19_FTX_H2	21021	23900	31689	86	13.9956
NABEC	NABEC_SH_04_21_FTX_H1	23501	26403	30894	115	16.9521
NABEC	NABEC_SH_04_21_FTX_H2	22827	24348	31089	131	21.193
NABEC	NABEC_SH_05_10_FTX_H1	21939	25265	28401	25	4.73405
NABEC	NABEC_SH_05_10_FTX_H2	18029	19740	25575	32	4.07843
NABEC	NABEC_SH_05_16_FTX_H1	20567	22905.5	27207	74	12.2118
NABEC	NABEC_SH_05_16_FTX_H2	20100	20654	28419	59	9.9782
NABEC	NABEC_SH_05_36_FTX_H1	19522	20873	31991	67	10.4215
NABEC	NABEC_SH_05_36_FTX_H2	16564	17358	25989	63	8.30395
NABEC	NABEC_SH_06_05_FTX_H1	17700	10926	32050	83	10.3287
NABEC	NABEC_SH_06_05_FTX_H2	19663	18789	31226	83	9.54067
**NABEC**	**NABEC_SH_06_25_FTX_H1**	**10931**	**5899.5**	**24866**	**34**	**3.71765**
**NABEC**	**NABEC_SH_06_25_FTX_H2**	**10285**	**4369**	**21158**	**28**	**3.14659**
NABEC	NABEC_SH_06_66_FTX_H1	26789	26909.5	33402	52	10.3793
NABEC	NABEC_SH_06_66_FTX_H2	25217	25187.5	31426	68	11.8792
NABEC	NABEC_SH_07_28_FTX_H1	25913	28225	30856	43	9.32408
NABEC	NABEC_SH_07_28_FTX_H2	22177	21777	34206	27	4.12851
NABEC	NABEC_SH_07_37_FTX_H1	25068	26192	36021	51	9.54767

NABEC	NABEC_SH_07_37_FTX_H2	24096	26061	31082	57	10.7478
NABEC	NABEC_SH_07_46_FTX_H1	22228	23037	29689	63	8.88675
NABEC	NABEC_SH_07_46_FTX_H2	22918	24723	31254	48	7.8298
NABEC	NABEC_SH_07_63_FTX_H1	25001	26718	31518	59	10.8581
NABEC	NABEC_SH_07_63_FTX_H2	29870	31959	37995	44	9.51772
NABEC	NABEC_SH_08_04_FTX_H1	26990	27984.5	33389	54	11.5657
NABEC	NABEC_SH_08_04_FTX_H2	25729	27635	31803	53	8.71446
NABEC	NABEC_SH_08_23_FTX_H1	18503	17763	28900	70	9.61583
NABEC	NABEC_SH_08_23_FTX_H2	17130	18481	28511	77	11.0255
NABEC	NABEC_SH_92_05_FTX_H1	20211	21794	29561	75	10.6035
NABEC	NABEC_SH_92_05_FTX_H2	19389	21967	29549	69	10.4732
NABEC	NABEC_SH_92_14_FTX_H1	23384	25116	30068	59	10.0905
NABEC	NABEC_SH_92_14_FTX_H2	25044	25666	30576	61	11.4969
NABEC	NABEC_SH_94_35_FTX_H1	22816	23243	30068	117	18.2906
NABEC	NABEC_SH_94_35_FTX_H2	21678	23269.5	31686	86	13.0079
NABEC	NABEC_SH_95_02_FTX_H1	16967	16635.5	26849	98	13.3653
NABEC	NABEC_SH_95_02_FTX_H2	18010	17289.5	29028	104	14.5775
NABEC	NABEC_SH_95_21_FTX_H1	15425	11082	29567	106	11.3117
NABEC	NABEC_SH_95_21_FTX_H2	19803	22257	33752	82	13.7495
NABEC	NABEC_SH_95_34_FTX_H1	23981	25477.5	31851	66	11.3455
NABEC	NABEC_SH_95_34_FTX_H2	24682	27640	31163	72	12.8733
NABEC	NABEC_SH_96_08_FTX_H1	25414	26769	29736	167	29.2776
NABEC	NABEC_SH_96_08_FTX_H2	24489	26122	29441	145	25.2421
NABEC	NABEC_SH_96_22_FTX_H1	20249	21673	31087	112	16.0672
NABEC	NABEC_SH_96_22_FTX_H2	22382	24244	32650	105	15.826
NABEC	NABEC_SH_96_30_FTX_H1	21060	21276	31202	94	15.7599
NABEC	NABEC_SH_96_30_FTX_H2	22680	25536	31413	94	14.7493
NABEC	NABEC_SH_96_32_FTX_H1	24685	27023	33261	87	13.483
NABEC	NABEC_SH_96_32_FTX_H2	22362	25296	33229	90	14.039
NABEC	NABEC_SH_96_35_FTX_H1	20534	22430	28830	79	13.5204
NABEC	NABEC_SH_96_35_FTX_H2	20635	24033	28886	77	13.7281
NABEC	NABEC_SH_96_38_FTX_H1	16349	13298	30463	91	10.8983
NABEC	NABEC_SH_96_38_FTX_H2	14074	8479	26477	111	12.146
NABEC	NABEC_SH_96_39_FTX_H1	18655	20146.5	26233	74	10.5087
NABEC	NABEC_SH_96_39_FTX_H2	19492	17414	28183	87	11.9706
NABEC	NABEC_SH_97_09_FTX_H1	12165	9621	22373	64	5.95009
NABEC	NABEC_SH_97_09_FTX_H2	11450	6029	24255	87	7.68159
NABEC	NABEC_SH_97_19_FTX_H1	14822	7469	28783	97	10.0421
NABEC	NABEC_SH_97_19_FTX_H2	16525	13105	29733	113	14.1491

NABEC	NABEC_SH_97_37_FTX_H1	21611	24834	30724	79	12.1224
NABEC	NABEC_SH_97_37_FTX_H2	20552	23036	30691	60	9.96352
NABEC	NABEC_SH_97_53_FTX_H1	21142	22971.5	31876	100	15.7973
NABEC	NABEC_SH_97_53_FTX_H2	22581	22587	29458	91	14.6381
NABEC	NABEC_SH_98_23_FTX_H1	24861	27735	32915	71	11.9039
NABEC	NABEC_SH_98_23_FTX_H2	23033	25664	29838	83	12.8961
NABEC	NABEC_SH_98_32_FTX_H1	20795	22062	28363	82	12.3789
NABEC	NABEC_SH_98_32_FTX_H2	19587	22587	31414	37	5.81389
NABEC	NABEC_SH_99_14_FTX_H1	21147	23259	26919	60	8.69897
NABEC	NABEC_SH_99_14_FTX_H2	21377	23561	30591	63	9.29212
NABEC	NABEC_SH_99_29_FTX_H1	20202	22554	27484	63	9.76977
NABEC	NABEC_SH_99_29_FTX_H2	22616	21588.5	29088	60	10.1266
NABEC	NABEC_SH_99_31_FTX_H1	23114	24107.5	33781	82	13.7509
NABEC	NABEC_SH_99_31_FTX_H2	23640	25804	30931	89	16.1258
NABEC	NABEC_SH_99_44_FTX_H1	22692	23374	28942	91	14.9677
NABEC	NABEC_SH_99_44_FTX_H2	21087	22780.5	27887	80	13.1078
NABEC	NABEC_SH_99_54_FTX_H1	14719	11602	29441	81	9.22995
NABEC	NABEC_SH_99_54_FTX_H2	15887	11084.5	30819	96	11.2608
NABEC	NABEC_UMARY_1013_FTX_H1	19592	21797.5	28596	66	10.1952
NABEC	NABEC_UMARY_1013_FTX_H2	15164	10979	28092	85	10.4187
NABEC	NABEC_UMARY_1027_FTX_H1	15264	15342.5	22486	86	11.6614
NABEC	NABEC_UMARY_1027_FTX_H2	17066	17602.5	26135	84	11.1986
NABEC	NABEC_UMARY_1028_FTX_H1	13133	5757.5	27437	92	7.40652
NABEC	NABEC_UMARY_1028_FTX_H2	14383	9945	26661	120	12.8972
NABEC	NABEC_UMARY_1064_FTX_H1	9270	6708	16323	312	22.473
NABEC	NABEC_UMARY_1064_FTX_H2	8416	6903	10659	349	28.3973
NABEC	NABEC_UMARY_1076_FTX_H1	17844	18431	25498	141	18.2496
NABEC	NABEC_UMARY_1076_FTX_H2	16612	15680	25861	130	15.8347
NABEC	NABEC_UMARY_1078_FTX_H1	14661	13300	22136	65	8.998
NABEC	NABEC_UMARY_1078_FTX_H2	19418	18865.5	24539	76	12.5049
NABEC	NABEC_UMARY_1079_FTX_H1	24406	25091	28826	41	7.54717
NABEC	NABEC_UMARY_1079_FTX_H2	26102	27425	30133	42	8.81991
NABEC	NABEC_UMARY_1101_FTX_H1	20550	20319	27316	139	19.8965
NABEC	NABEC_UMARY_1101_FTX_H2	22104	23338	27910	127	22.2083
NABEC	NABEC_UMARY_1104_FTX_H1	17803	17486.5	27014	102	12.8347
NABEC	NABEC_UMARY_1104_FTX_H2	16679	14598	25214	109	13.5594
NABEC	NABEC_UMARY_1113_FTX_H1	16737	14608	28882	88	11.4446
NABEC	NABEC_UMARY_1113_FTX_H2	13301	10446.5	23742	60	7.22403
NABEC	NABEC_UMARY_1114_FTX_H1	18044	16872	29769	89	12.504

NABEC	NABEC_UMARY_1114_FTX_H2	18273	18490	27258	88	11.3505
NABEC	NABEC_UMARY_1133_FTX_H1	24272	24767	29724	73	12.2368
NABEC	NABEC_UMARY_1133_FTX_H2	20982	22366	26761	43	9.02163
NABEC	NABEC_UMARY_1134_FTX_H1	18830	19479	28053	69	9.32729
NABEC	NABEC_UMARY_1134_FTX_H2	20883	23299.5	28851	66	9.65426
NABEC	NABEC_UMARY_1135_FTX_H1	20311	24473	29981	66	9.91611
NABEC	NABEC_UMARY_1135_FTX_H2	21613	25606	29008	80	14.5991
NABEC	NABEC_UMARY_1136_FTX_H1	17381	17488	27676	82	10.9572
NABEC	NABEC_UMARY_1136_FTX_H2	15560	13895	24540	93	10.376
NABEC	NABEC_UMARY_1158_FTX_H1	18212	18846	29229	67	10.8065
NABEC	NABEC_UMARY_1158_FTX_H2	17545	18727	25584	58	8.42975
NABEC	NABEC_UMARY_1259_FTX_H1	18529	16082	28883	93	12.4999
NABEC	NABEC_UMARY_1259_FTX_H2	17052	17562	25432	104	13.0623
NABEC	NABEC_UMARY_1266_FTX_H1	10973	7533	22692	113	10.5448
NABEC	NABEC_UMARY_1266_FTX_H2	13436	9248.5	25825	122	12.654
NABEC	NABEC_UMARY_1277_FTX_H1	15348	13129	27867	159	15.5415
NABEC	NABEC_UMARY_1277_FTX_H2	14160	11603.5	25025	128	14.3363
NABEC	NABEC_UMARY_1279_FTX_H1	19028	21710.5	30795	142	18.8693
NABEC	NABEC_UMARY_1279_FTX_H2	19254	19126	31572	144	19.0495
NABEC	NABEC_UMARY_1322_FTX_H1	21482	24479	27189	63	11.6192
NABEC	NABEC_UMARY_1322_FTX_H2	19921	21191.5	27916	64	10.5039
NABEC	NABEC_UMARY_1323_FTX_H1	20604	23177.5	27762	76	11.9348
NABEC	NABEC_UMARY_1323_FTX_H2	18125	17579	27999	81	11.652
NABEC	NABEC_UMARY_1326_FTX_H1	14158	10704.5	24990	132	12.8795
NABEC	NABEC_UMARY_1326_FTX_H2	12913	8238	24125	131	13.6651
NABEC	NABEC_UMARY_1347_FTX_H1	19203	20665	28681	73	9.34174
NABEC	NABEC_UMARY_1347_FTX_H2	15807	14774	28718	93	11.7325
NABEC	NABEC_UMARY_1362_FTX_H1	10919	4997	27458	148	13.054
NABEC	NABEC_UMARY_1362_FTX_H2	11772	7404.5	23965	182	17.2638
NABEC	NABEC_UMARY_1363_FTX_H1	22140	22133	30972	71	10.0723
NABEC	NABEC_UMARY_1363_FTX_H2	20439	19428	29072	67	10.5332
NABEC	NABEC_UMARY_1378_FTX_H1	17577	18140	28268	66	9.27494
NABEC	NABEC_UMARY_1378_FTX_H2	17244	18185	26765	70	9.45306
NABEC	NABEC_UMARY_1379_FTX_H1	17271	18465	24671	69	8.85105
NABEC	NABEC_UMARY_1379_FTX_H2	20462	20692	26366	65	10.1065
NABEC	NABEC_UMARY_1406_FTX_H1	15084	10560.5	22043	166	18.9231
NABEC	NABEC_UMARY_1406_FTX_H2	14888	9065	27114	125	13.5787
NABEC	NABEC_UMARY_1410_FTX_H1	17336	17062	25542	92	13.0601
NABEC	NABEC_UMARY_1410_FTX_H2	21170	20548	29344	102	16.1719

NABEC	NABEC_UMARY_1442_FTX_H1	16963	14099	30543	119	13.6583
NABEC	NABEC_UMARY_1442_FTX_H2	17392	16127	29531	117	17.3621
NABEC	NABEC_UMARY_1454_FTX_H1	10921	4796	25868	120	11.0529
NABEC	NABEC_UMARY_1454_FTX_H2	11391	5028	25198	105	10.6266
NABEC	NABEC_UMARY_1455_FTX_H1	23670	25505.5	32631	40	6.47877
NABEC	NABEC_UMARY_1455_FTX_H2	23069	22635	32355	46	7.82451
NABEC	NABEC_UMARY_1461_FTX_H1	20481	19853	26596	75	12.9779
NABEC	NABEC_UMARY_1461_FTX_H2	24203	25088	27156	69	12.02
NABEC	NABEC_UMARY_1464_FTX_H1	18564	19134	29244	76	10.9873
NABEC	NABEC_UMARY_1464_FTX_H2	21219	21997	29557	93	11.7414
NABEC	NABEC_UMARY_1465_FTX_H1	11476	8156.5	12403	254	22.1029
NABEC	NABEC_UMARY_1465_FTX_H2	11542	8108.5	12726	256	21.0232
NABEC	NABEC_UMARY_1475_FTX_H1	18379	15871	30603	82	12.5218
NABEC	NABEC_UMARY_1475_FTX_H2	20549	21178	28239	85	12.5825
NABEC	NABEC_UMARY_1486_FTX_H1	18314	16380.5	30649	68	10.4343
NABEC	NABEC_UMARY_1486_FTX_H2	18727	19180	29242	71	9.52023
NABEC	NABEC_UMARY_1496_FTX_H1	22461	24867	33944	73	10.9329
NABEC	NABEC_UMARY_1496_FTX_H2	20245	20630.5	27854	80	13.2975
NABEC	NABEC_UMARY_1498_FTX_H1	16290	13702	27334	123	15.3956
NABEC	NABEC_UMARY_1498_FTX_H2	17023	14475.5	29567	108	14.4591
NABEC	NABEC_UMARY_1535_FTX_H1	16339	15737	30938	98	11.7432
NABEC	NABEC_UMARY_1535_FTX_H2	14718	11497.5	27294	100	11.8462
NABEC	NABEC_UMARY_1539_FTX_H1	20196	22035	30918	76	10.7507
NABEC	NABEC_UMARY_1539_FTX_H2	18747	21450.5	28380	76	10.5791
NABEC	NABEC_UMARY_1540_FTX_H1	16941	16078	24810	103	14.0784
NABEC	NABEC_UMARY_1540_FTX_H2	15985	13334.5	25638	88	11.1121
NABEC	NABEC_UMARY_1541_FTX_H1	17019	14773	28252	107	13.9457
NABEC	NABEC_UMARY_1541_FTX_H2	16809	15469.5	29331	88	11.8169
NABEC	NABEC_UMARY_1543_FTX_H1	17558	18873.5	26570	114	13.726
NABEC	NABEC_UMARY_1543_FTX_H2	15438	12513	27136	135	14.6549
NABEC	NABEC_UMARY_1545_FTX_H1	13718	7782.5	26063	158	15.012
NABEC	NABEC_UMARY_1545_FTX_H2	12706	6405	25429	158	14.4107
NABEC	NABEC_UMARY_1570_FTX_H1	22386	23178	31631	68	11.5735
NABEC	NABEC_UMARY_1570_FTX_H2	23823	25820	31715	55	8.81575
NABEC	NABEC_UMARY_1584_FTX_H1	20749	21582.5	32364	74	10.6271
NABEC	NABEC_UMARY_1584_FTX_H2	20901	23292.5	29873	88	13.3248
NABEC	NABEC_UMARY_1607_FTX_H1	16354	13762.5	26133	120	13.8586
NABEC	NABEC_UMARY_1607_FTX_H2	17292	17417	27301	131	15.9395
NABEC	NABEC_UMARY_1609_FTX_H1	17839	17223	22363	55	8.71528

NABEC	NABEC_UMARY_1609_FTX_H2	19100	20177	27171	85	11.6279
NABEC	NABEC_UMARY_1648_FTX_H1	20662	23417	33137	64	10.1433
NABEC	NABEC_UMARY_1648_FTX_H2	22408	23085	37508	51	9.76282
NABEC	NABEC_UMARY_165_FTX_H1	21168	22642.5	28896	106	17.1248
NABEC	NABEC_UMARY_165_FTX_H2	17472	16734	26733	111	14.6688
NABEC	NABEC_UMARY_1668_FTX_H1	15381	12095	25459	89	11.0913
NABEC	NABEC_UMARY_1668_FTX_H2	17131	14067	30722	99	12.5714
NABEC	NABEC_UMARY_1672_FTX_H1	21705	25167	32558	71	11.2019
NABEC	NABEC_UMARY_1672_FTX_H2	17282	18297	26296	74	11.397
NABEC	NABEC_UMARY_1675_FTX_H1	21852	24242	30746	77	12.84
NABEC	NABEC_UMARY_1675_FTX_H2	24542	24034	32889	66	12.3011
NABEC	NABEC_UMARY_1710_FTX_H1	15722	13784.5	23634	92	11.7564
NABEC	NABEC_UMARY_1710_FTX_H2	17615	16804	28158	70	8.5305
NABEC	NABEC_UMARY_1713_FTX_H1	24234	25197	30843	65	12.4639
NABEC	NABEC_UMARY_1713_FTX_H2	22644	23844	31284	78	12.6982
NABEC	NABEC_UMARY_1743_FTX_H1	21082	21611.5	29456	90	13.4732
NABEC	NABEC_UMARY_1743_FTX_H2	16225	16964	24857	96	12.8227
NABEC	NABEC_UMARY_177_FTX_H1	23271	25121	30518	76	12.7589
NABEC	NABEC_UMARY_177_FTX_H2	26141	26427	31825	81	14.5463
NABEC	NABEC_UMARY_1795_FTX_H1	10775	7916	10956	194	15.6175
NABEC	NABEC_UMARY_1795_FTX_H2	10596	7831	11025	170	13.5087
NABEC	NABEC_UMARY_1796_FTX_H1	20019	17260	30706	63	9.48621
NABEC	NABEC_UMARY_1796_FTX_H2	24602	25638	31506	58	11.0367
NABEC	NABEC_UMARY_1797_FTX_H1	19249	18758	28027	61	8.66035
NABEC	NABEC_UMARY_1797_FTX_H2	21943	22266	28204	71	12.3541
NABEC	NABEC_UMARY_1823_FTX_H1	9245	3610	25728	200	14.2349
NABEC	NABEC_UMARY_1823_FTX_H2	10388	3736	25582	175	15.3668
NABEC	NABEC_UMARY_1825_FTX_H1	31373	32379	33853	19	5.31617
NABEC	NABEC_UMARY_1825_FTX_H2	31239	31848.5	34593	34	8.55042
NABEC	NABEC_UMARY_1827_FTX_H1	17355	18457.5	26270	98	12.7671
NABEC	NABEC_UMARY_1827_FTX_H2	18920	19483	26412	113	14.9784
NABEC	NABEC_UMARY_1841_FTX_H1	15156	14396	24975	130	15.8322
NABEC	NABEC_UMARY_1841_FTX_H2	12703	8992.5	24864	130	12.7683
NABEC	NABEC_UMARY_1843_FTX_H1	21455	24077	32242	81	13.5953
NABEC	NABEC_UMARY_1843_FTX_H2	20057	19756.5	31329	96	13.3937
NABEC	NABEC_UMARY_1845_FTX_H1	23271	25639.5	30726	64	10.661
NABEC	NABEC_UMARY_1845_FTX_H2	21455	22423	30357	77	10.5272
NABEC	NABEC_UMARY_1847_FTX_H1	12870	10917	24715	134	14.2286
NABEC	NABEC_UMARY_1847_FTX_H2	11996	7521.5	23125	120	11.7895

NABEC	NABEC_UMARY_1849_FTX_H1	12140	9643.5	23344	128	14.8217
NABEC	NABEC_UMARY_1849_FTX_H2	11668	7434	22260	129	11.2388
NABEC	NABEC_UMARY_1861_FTX_H1	24207	24973	30927	81	14.1219
NABEC	NABEC_UMARY_1861_FTX_H2	20257	21335	26118	56	10.3047
NABEC	NABEC_UMARY_1862_FTX_H1	18582	19093	29164	95	13.7323
NABEC	NABEC_UMARY_1862_FTX_H2	16460	13498	27277	68	8.64435
NABEC	NABEC_UMARY_1865_FTX_H1	12336	10278	23870	125	12.3963
NABEC	NABEC_UMARY_1865_FTX_H2	12195	8636	24249	164	15.1352
NABEC	NABEC_UMARY_1909_FTX_H1	9493	6339	16798	351	23.0051
NABEC	NABEC_UMARY_1909_FTX_H2	8990	6412	11252	323	19.2477
NABEC	NABEC_UMARY_1935_FTX_H1	14376	12035	25901	79	8.76495
NABEC	NABEC_UMARY_1935_FTX_H2	17907	15208	30462	75	9.79053
NABEC	NABEC_UMARY_240_FTX_H1	17204	16354	23693	41	5.5927
NABEC	NABEC_UMARY_240_FTX_H2	17976	16375	22155	52	6.66737
NABEC	NABEC_UMARY_251_FTX_H1	17140	19440	24437	45	6.66544
NABEC	NABEC_UMARY_251_FTX_H2	18919	18774	26661	63	9.91831
NABEC	NABEC_UMARY_26_FTX_H1	15625	12334.5	28035	78	10.2595
NABEC	NABEC_UMARY_26_FTX_H2	14461	10726	26715	74	7.42046
NABEC	NABEC_UMARY_288_FTX_H1	14186	9932	27390	97	10.0893
NABEC	NABEC_UMARY_288_FTX_H2	13011	9300	24877	105	9.73447
NABEC	NABEC_UMARY_4228_FTX_H1	13107	11772	23155	108	10.4163
NABEC	NABEC_UMARY_4228_FTX_H2	13367	10496	23879	75	7.8332
NABEC	NABEC_UMARY_4263_FTX_H1	12342	7717	24754	190	15.8851
NABEC	NABEC_UMARY_4263_FTX_H2	12004	7615	23805	189	16.4465
NABEC	NABEC_UMARY_4540_FTX_H1	17109	16911	28277	93	12.2541
NABEC	NABEC_UMARY_4540_FTX_H2	19726	21713	29568	65	11.4748
NABEC	NABEC_UMARY_4542_FTX_H1	16067	11185	28815	118	12.7466
NABEC	NABEC_UMARY_4542_FTX_H2	18024	18269	29810	95	12.302
NABEC	NABEC_UMARY_4546_FTX_H1	21628	21065	33426	74	11.3136
NABEC	NABEC_UMARY_4546_FTX_H2	24191	24972	31678	71	13.5337
NABEC	NABEC_UMARY_4549_FTX_H1	13445	10293	23318	86	9.14015
NABEC	NABEC_UMARY_4549_FTX_H2	12422	8834.5	21422	114	11.2985
NABEC	NABEC_UMARY_455_FTX_H1	17428	15750	30537	97	12.304
NABEC	NABEC_UMARY_455_FTX_H2	17279	13262	29942	83	10.2537
NABEC	NABEC_UMARY_4592_FTX_H1	12896	9482.5	24750	136	13.922
NABEC	NABEC_UMARY_4592_FTX_H2	11639	7474	22111	145	12.7267
NABEC	NABEC_UMARY_4636_FTX_H1	20074	20361	30987	61	9.26266
NABEC	NABEC_UMARY_4636_FTX_H2	18027	20541	28517	64	9.07636
NABEC	NABEC_UMARY_4640_FTX_H1	22772	21638.5	31170	56	10.217

NABEC	NABEC_UMARY_4640_FTX_H2	25325	27524	31459	50	10.5073
NABEC	NABEC_UMARY_4669_FTX_H1	24138	26762	32124	45	8.53466
NABEC	NABEC_UMARY_4669_FTX_H2	23575	24516	29888	58	10.552
NABEC	NABEC_UMARY_4725_FTX_H1	18896	15593.5	30748	82	10.8968
NABEC	NABEC_UMARY_4725_FTX_H2	21620	20814	28351	57	8.75789
NABEC	NABEC_UMARY_4726_FTX_H1	20224	22494	28389	107	15.7835
NABEC	NABEC_UMARY_4726_FTX_H2	21287	23378	27504	102	15.715
NABEC	NABEC_UMARY_4727_FTX_H1	23102	24133	31894	85	13.5645
NABEC	NABEC_UMARY_4727_FTX_H2	19722	21032	28996	59	9.71722
NABEC	NABEC_UMARY_4728_FTX_H1	16138	14281	25635	135	15.448
NABEC	NABEC_UMARY_4728_FTX_H2	14212	9291	28600	113	11.5366
NABEC	NABEC_UMARY_4729_FTX_H1	18141	17072	27009	68	8.70879
NABEC	NABEC_UMARY_4729_FTX_H2	18877	18038	29956	75	10.7097
NABEC	NABEC_UMARY_4735_FTX_H1	16482	16284.5	27122	48	6.54291
NABEC	NABEC_UMARY_4735_FTX_H2	16834	12221	34645	55	7.30466
NABEC	NABEC_UMARY_4782_FTX_H1	24800	23532	32076	74	12.8565
NABEC	NABEC_UMARY_4782_FTX_H2	24468	27279	33787	66	11.0681
NABEC	NABEC_UMARY_4786_FTX_H1	20072	21056	32327	68	9.85166
NABEC	NABEC_UMARY_4786_FTX_H2	17743	21237.5	28816	42	6.07244
NABEC	NABEC_UMARY_4789_FTX_H1	26891	27512	31989	51	10.1933
NABEC	NABEC_UMARY_4789_FTX_H2	24582	27595.5	31887	44	8.48534
NABEC	NABEC_UMARY_4903_FTX_H1	20468	21944	24469	69	10.7885
NABEC	NABEC_UMARY_4903_FTX_H2	19063	20209	24167	80	12.7962
NABEC	NABEC_UMARY_4906_FTX_H1	21416	24410	29672	75	11.8473
NABEC	NABEC_UMARY_4906_FTX_H2	19603	21198.5	29871	82	12.5153
NABEC	NABEC_UMARY_4916_FTX_H1	23470	25572	30273	65	11.2773
NABEC	NABEC_UMARY_4916_FTX_H2	21098	22382	27197	63	9.38105
NABEC	NABEC_UMARY_4924_FTX_H1	20190	24676.5	31388	84	12.638
NABEC	NABEC_UMARY_4924_FTX_H2	20876	25300	29296	81	13.8512
NABEC	NABEC_UMARY_5024_FTX_H1	14205	13008	21470	133	16.4704
NABEC	NABEC_UMARY_5024_FTX_H2	14187	13376	22789	123	15.1032
NABEC	NABEC_UMARY_5028_FTX_H1	14792	13128	22120	109	13.6357
NABEC	NABEC_UMARY_5028_FTX_H2	15612	13317	24367	116	14.18
NABEC	NABEC_UMARY_5077_FTX_H1	18608	18978	28852	65	8.48306
NABEC	NABEC_UMARY_5077_FTX_H2	16753	14416	27595	63	8.34443
NABEC	NABEC_UMARY_5078_FTX_H1	13120	10659	25851	125	13.4212
NABEC	NABEC_UMARY_5078_FTX_H2	12988	8874	25320	116	11.3519
NABEC	NABEC_UMARY_5079_FTX_H1	16380	16765.5	22003	106	13.5917
NABEC	NABEC_UMARY_5079_FTX_H2	15291	15118	23761	98	12.3852

NABEC	NABEC_UMARY_5086_FTX_H1	20427	22823.5	30458	106	15.7961
NABEC	NABEC_UMARY_5086_FTX_H2	16271	12725	29569	87	10.1377
NABEC	NABEC_UMARY_5087_FTX_H1	14255	11772	25601	98	10.8556
NABEC	NABEC_UMARY_5087_FTX_H2	15318	7613.5	29793	96	10.3972
NABEC	NABEC_UMARY_5088_FTX_H1	23389	27811	32687	72	12.3262
NABEC	NABEC_UMARY_5088_FTX_H2	25511	27835	32733	67	12.2972
NABEC	NABEC_UMARY_5114_FTX_H1	11603	7187	24491	65	6.07383
NABEC	NABEC_UMARY_5114_FTX_H2	13368	11218	25667	72	9.05127
NABEC	NABEC_UMARY_5117_FTX_H1	9457	3269	28525	90	6.5279
NABEC	NABEC_UMARY_5117_FTX_H2	11690	4675	29267	79	7.51405
NABEC	NABEC_UMARY_5120_FTX_H1	20789	21726	30485	108	15.5184
NABEC	NABEC_UMARY_5120_FTX_H2	18902	16619.5	26524	94	13.6327
NABEC	NABEC_UMARY_5123_FTX_H1	20878	21384	29783	77	11.665
NABEC	NABEC_UMARY_5123_FTX_H2	21103	22692	29720	74	10.7859
NABEC	NABEC_UMARY_5171_FTX_H1	14601	9298	26847	71	7.62842
NABEC	NABEC_UMARY_5171_FTX_H2	15147	13401	23914	76	8.11693
NABEC	NABEC_UMARY_5179_FTX_H1	13369	10884	23290	111	10.5044
NABEC	NABEC_UMARY_5179_FTX_H2	14018	8063	29641	110	12.2607
NABEC	NABEC_UMARY_544_FTX_H1	16650	14882	27782	206	24.2635
NABEC	NABEC_UMARY_544_FTX_H2	16598	15719	27998	177	21.1518
NABEC	NABEC_UMARY_55_FTX_H1	14810	12666	23899	215	24.0843
NABEC	NABEC_UMARY_55_FTX_H2	15263	13051	25572	195	16.8109
NABEC	NABEC_UMARY_602_FTX_H1	17338	18963	27924	107	13.5669
NABEC	NABEC_UMARY_602_FTX_H2	14542	9769	27250	101	11.7374
NABEC	NABEC_UMARY_604_FTX_H1	20031	18292	31541	74	12.6
NABEC	NABEC_UMARY_604_FTX_H2	17267	14236	26600	95	13.16
NABEC	NABEC_UMARY_650_FTX_H1	18955	17742	29078	88	14.4182
NABEC	NABEC_UMARY_650_FTX_H2	18471	16104.5	29527	84	12.1581
NABEC	NABEC_UMARY_671_FTX_H1	19279	21027.5	29935	62	9.30857
NABEC	NABEC_UMARY_671_FTX_H2	22305	24775	35345	61	10.519
NABEC	NABEC_UMARY_794_FTX_H1	21348	22823	28521	56	9.11343
NABEC	NABEC_UMARY_794_FTX_H2	20088	22691	27249	69	11.709
NABEC	NABEC_UMARY_813_FTX_H1	16863	17013	25288	71	8.69933
NABEC	NABEC_UMARY_813_FTX_H2	18235	21385	27328	82	11.6362
NABEC	NABEC_UMARY_818_FTX_H1	23860	24129	32343	61	9.92742
NABEC	NABEC_UMARY_818_FTX_H2	22133	23840	31404	55	9.73486
NABEC	NABEC_UMARY_819_FTX_H1	19448	20753	29139	73	11.1681
NABEC	NABEC_UMARY_819_FTX_H2	23534	24761	31156	63	10.7102
NABEC	NABEC_UMARY_914_FTX_H1	16940	16791.5	25289	96	12.3138

NABEC	NABEC_UMARY_914_FTX_H2	12646	8154	25142	83	8.24315
NABEC	NABEC_UMARY_933_FTX_H1	14521	13426.5	24102	122	13.0265
NABEC	NABEC_UMARY_933_FTX_H2	17976	18188	26128	114	15.4733
	Avg	18576.0075	17994.03	28141.32	92.26	12.11920033
	Stdv	4142.022821	6333.681124	3836.136741	42.48290715	3.568926177
HBCC	HBCC_81925_FTX_H1	11887	6579	24127	171	15.7278
HBCC	HBCC_81925_FTX_H2	10454	3945	26066	183	13.9263
HBCC	HBCC_81927_FTX_H1	17219	17963	23056	65	8.87095
HBCC	HBCC_81927_FTX_H2	15346	13515	25817	63	8.18201
HBCC	HBCC_81928_FTX_H1	17202	11297.5	35283	66	7.40996
HBCC	HBCC_81928_FTX_H2	16447	12285	30441	55	8.13222
HBCC	HBCC_81929_FTX_H1	18938	19251	29150	59	9.87842
HBCC	HBCC_81929_FTX_H2	20585	18973.5	32002	46	7.87996
HBCC	HBCC_81930_FTX_H1	23071	25263	31951	75	13.2503
HBCC	HBCC_81930_FTX_H2	21318	23757	29544	77	11.5576
HBCC	HBCC_81931_FTX_H1	23597	27481	35444	79	13.7373
HBCC	HBCC_81931_FTX_H2	22717	20784	33913	58	10.3601
HBCC	HBCC_81932_FTX_H1	10858	5075	24072	101	8.86501
HBCC	HBCC_81932_FTX_H2	9209	5480	18282	86	7.51939
HBCC	HBCC_81934_FTX_H1	18834	16419	31246	76	10.5986
HBCC	HBCC_81934_FTX_H2	21624	20469	34696	84	12.126
HBCC	HBCC_81936_FTX_H1	22768	24328.5	34445	64	10.0625
HBCC	HBCC_81936_FTX_H2	22404	24750	33175	79	11.8178
HBCC	HBCC_81937_FTX_H1	8622	5841	16045	104	8.12543
HBCC	HBCC_81937_FTX_H2	10526	5738	20515	127	10.7595
HBCC	HBCC_81939_FTX_H1	8633	5800.5	14186	126	8.84005
HBCC	HBCC_81939_FTX_H2	7116	4426	12382	165	10.6916
HBCC	HBCC_81941_FTX_H1	19314	18712.5	29567	84	13.7611
HBCC	HBCC_81941_FTX_H2	18269	15532	33306	91	10.9632
HBCC	HBCC_81943_FTX_H1	9593	6377	13492	110	9.32314
HBCC	HBCC_81943_FTX_H2	6578	4813	9417	139	8.41522
HBCC	HBCC_81944_FTX_H1	16991	13615.5	29166	116	13.1197
HBCC	HBCC_81944_FTX_H2	15946	9685	31724	142	14.7163
HBCC	HBCC_81945_FTX_H1	17238	12285	34879	85	10.3558
HBCC	HBCC_81945_FTX_H2	18971	13147.5	38625	78	10.5785
HBCC	HBCC_81946_FTX_H1	15439	10984	28453	94	10.4992
HBCC	HBCC_81946_FTX_H2	13081	5597.5	29452	96	12.0468

HBCC	HBCC_81948_FTX_H1	9691	6816	17915	85	6.26528
HBCC	HBCC_81948_FTX_H2	10687	7108.5	18732	78	6.37034
HBCC	HBCC_81949_FTX_H1	17959	17279	25310	71	9.36402
HBCC	HBCC_81949_FTX_H2	13951	11054	22286	61	7.31395
HBCC	HBCC_81950_FTX_H1	17524	16747	28779	75	10.3161
HBCC	HBCC_81950_FTX_H2	12922	7890	27908	71	8.66087
HBCC	HBCC_81951_FTX_H1	8459	2982	23684	146	8.99179
HBCC	HBCC_81951_FTX_H2	8343	3307	21943	162	12.8449
HBCC	HBCC_81952_FTX_H1	12165	8656	25495	159	14.7689
HBCC	HBCC_81952_FTX_H2	12385	7977	25650	129	13.3118
HBCC	HBCC_81953_FTX_H1	12073	5151.5	31092	114	9.97203
HBCC	HBCC_81953_FTX_H2	11856	4905	27198	115	9.73026
HBCC	HBCC_81954_FTX_H1	8706	3366	19986	171	13.8403
HBCC	HBCC_81954_FTX_H2	9294	3623.5	22657	164	12.3978
HBCC	HBCC_81955_FTX_H1	6345	1307	24923	203	11.243
HBCC	HBCC_81955_FTX_H2	6629	1542	23062	197	11.9173
HBCC	HBCC_81956_FTX_H1	11819	4503.5	27481	140	11.7892
HBCC	HBCC_81956_FTX_H2	11191	3523	27281	175	14.6055
HBCC	HBCC_81957_FTX_H1	4147	1370	13671	273	10.5757
HBCC	HBCC_81957_FTX_H2	4477	1227	17157	255	9.69042
HBCC	HBCC_81958_FTX_H1	12864	7151	27954	81	8.33515
HBCC	HBCC_81958_FTX_H2	11672	7024	25727	99	8.67752
HBCC	HBCC_81959_FTX_H1	15485	10754	30649	129	14.5086
HBCC	HBCC_81959_FTX_H2	18549	14121	29001	113	14.0411
HBCC	HBCC_81960_FTX_H1	15759	14079	27613	122	14.668
HBCC	HBCC_81960_FTX_H2	16721	16269	27463	107	12.2458
HBCC	HBCC_81961_FTX_H1	15227	12267.5	26687	118	15.0364
HBCC	HBCC_81961_FTX_H2	11957	7413	23367	112	11.189
HBCC	HBCC_81962_FTX_H1	14749	9621	28448	127	14.2961
HBCC	HBCC_81962_FTX_H2	17067	12992	26980	108	14.9216
HBCC	HBCC_81964_FTX_H1	10463	3798	30313	153	12.5518
HBCC	HBCC_81964_FTX_H2	7626	2074.5	23180	176	10.8688
HBCC	HBCC_81965_FTX_H1	13568	10804	23416	47	5.21983
HBCC	HBCC_81965_FTX_H2	19561	21155	30519	63	9.10774
HBCC	HBCC_81967_FTX_H1	18346	17920.5	28569	118	15.6039
HBCC	HBCC_81967_FTX_H2	18292	19508	28628	109	13.5523
HBCC	HBCC_81968_FTX_H1	10940	6590	23153	121	9.90635
HBCC	HBCC_81968_FTX_H2	14230	10519	27847	137	13.4309
HBCC	HBCC_81969_FTX_H1	14278	9290	28908	98	10.6915

HBCC	HBCC_81969_FTX_H2	11740	7530	21137	93	8.63904
HBCC	HBCC_81970_FTX_H1	19999	23523	28546	59	9.41269
HBCC	HBCC_81970_FTX_H2	16100	14809	26389	73	10.4403
HBCC	HBCC_81971_FTX_H1	11852	5782	28546	101	8.51957
HBCC	HBCC_81971_FTX_H2	13759	7078	30107	109	10.1591
HBCC	HBCC_81972_FTX_H1	15244	10566.5	26903	100	10.9807
HBCC	HBCC_81972_FTX_H2	12589	7134.5	26230	90	8.69802
HBCC	HBCC_81973_FTX_H1	9084	3637	26327	178	11.6738
HBCC	HBCC_81973_FTX_H2	10015	6038.5	20329	182	16.6538
HBCC	HBCC_81974_FTX_H1	16495	13503	33082	90	11.6175
HBCC	HBCC_81974_FTX_H2	16966	12209.5	32113	96	11.8617
HBCC	HBCC_81975_FTX_H1	8931	5542	14965	199	13.4922
HBCC	HBCC_81975_FTX_H2	8867	5659	14559	214	14.5009
HBCC	HBCC_81976_FTX_H1	8453	6904	13811	167	11.7978
HBCC	HBCC_81976_FTX_H2	10465	9179	14893	139	11.9095
HBCC	HBCC_81977_FTX_H1	14545	9456.5	27598	86	9.68784
HBCC	HBCC_81977_FTX_H2	16346	14955	27830	64	7.50214
HBCC	HBCC_81978_FTX_H1	20578	20470.5	27289	68	10.7472
HBCC	HBCC_81978_FTX_H2	17847	18307	22080	74	10.9495
HBCC	HBCC_81979_FTX_H1	8352	1920	26046	220	13.8436
HBCC	HBCC_81979_FTX_H2	8737	2151	29949	181	12.4445
HBCC	HBCC_81980_FTX_H1	8616	4009.5	17806	144	11.6256
HBCC	HBCC_81980_FTX_H2	11443	8446	21527	132	13.1975
HBCC	HBCC_81981_FTX_H1	20577	18981	28694	88	12.4998
HBCC	HBCC_81981_FTX_H2	18129	15354	29097	93	10.8675
HBCC	HBCC_81982_FTX_H1	9220	5519	19645	93	6.77468
HBCC	HBCC_81982_FTX_H2	10810	6775	19115	94	9.38781
HBCC	HBCC_81983_FTX_H1	16064	13442.5	28435	98	11.5054
HBCC	HBCC_81983_FTX_H2	17860	17212	30614	61	6.76446
HBCC	HBCC_81984_FTX_H1	18512	20533.5	25720	70	9.70044
HBCC	HBCC_81984_FTX_H2	16838	16229	23952	65	8.25937
HBCC	HBCC_81985_FTX_H1	20674	22784	28755	57	8.93807
HBCC	HBCC_81985_FTX_H2	15199	16209	21246	53	7.44109
HBCC	HBCC_81986_FTX_H1	5522	1272.5	25410	172	7.31517
HBCC	HBCC_81986_FTX_H2	7134	1309	30892	218	10.6689
HBCC	HBCC_81987_FTX_H1	3607	1201	18853	526	13.2443
HBCC	HBCC_81987_FTX_H2	3833	1268	22192	512	14.8306
HBCC	HBCC_81988_FTX_H1	9706	3064.5	26865	156	10.5078
HBCC	HBCC_81988_FTX_H2	9727	3867	26428	148	9.6958

HBCC	HBCC_81989_FTX_H1	16691	14453	27420	100	11.2973
HBCC	HBCC_81989_FTX_H2	18730	18972	29064	91	11.9944
HBCC	HBCC_81990_FTX_H1	15345	11520.5	27324	96	10.2694
HBCC	HBCC_81990_FTX_H2	13246	6381.5	28797	90	9.82788
HBCC	HBCC_81991_FTX_H1	21833	23189	31269	96	13.3573
HBCC	HBCC_81991_FTX_H2	18852	18416	30387	86	10.7855
HBCC	HBCC_81992_FTX_H1	15856	13151.5	29291	72	8.45567
HBCC	HBCC_81992_FTX_H2	12886	11801	25791	53	5.73226
HBCC	HBCC_81993_FTX_H1	16552	16143.5	25798	112	14.2704
HBCC	HBCC_81993_FTX_H2	19832	19013.5	26313	98	15.7854
HBCC	HBCC_81995_FTX_H1	12419	9894	20578	72	7.83533
HBCC	HBCC_81995_FTX_H2	11574	5876	24047	78	7.87243
HBCC	HBCC_81996_FTX_H1	6800	1681.5	20697	180	11.0704
HBCC	HBCC_81996_FTX_H2	8005	2299	22601	185	12.7437
HBCC	HBCC_81997_FTX_H1	6880	2262	21581	243	11.7768
HBCC	HBCC_81997_FTX_H2	7266	2374.5	24166	238	12.0923
HBCC	HBCC_81998_FTX_H1	6844	1828	23090	132	8.78534
HBCC	HBCC_81998_FTX_H2	5596	1566	23983	137	6.01117
HBCC	HBCC_81999_FTX_H1	4972	2685.5	9655	262	10.1926
HBCC	HBCC_81999_FTX_H2	4894	2763.5	9775	258	11.6888
HBCC	HBCC_82002_FTX_H1	7968	5070	13232	175	11.7381
HBCC	HBCC_82002_FTX_H2	9746	7896	16368	187	15.8705
HBCC	HBCC_82003_FTX_H1	11716	5862	25281	107	10.2341
HBCC	HBCC_82003_FTX_H2	10898	5686	26040	95	8.33211
HBCC	HBCC_82004_FTX_H1	13344	7877	28622	89	9.28453
HBCC	HBCC_82004_FTX_H2	10743	5121	23788	95	9.07839
HBCC	HBCC_82005_FTX_H1	17658	17118	33136	83	10.651
HBCC	HBCC_82005_FTX_H2	16593	15301.5	26890	78	10.156
HBCC	HBCC_82006_FTX_H1	15218	13382	26131	124	14.9532
HBCC	HBCC_82006_FTX_H2	12214	10194.5	20824	128	11.6659
HBCC	HBCC_82007_FTX_H1	7312	4664	13721	183	11.0872
HBCC	HBCC_82007_FTX_H2	7797	4320	15191	164	10.9355
HBCC	HBCC_82008_FTX_H1	12255	7404	25190	143	12.5597
HBCC	HBCC_82008_FTX_H2	12182	6943	26292	135	10.4797
HBCC	HBCC_82009_FTX_H1	9300	7204	16396	177	14.9334
HBCC	HBCC_82009_FTX_H2	8455	6613	14446	171	14.6981
HBCC	HBCC_82010_FTX_H1	13802	11602	23607	147	16.52
HBCC	HBCC_82010_FTX_H2	13232	10217.5	22061	110	12.3995
HBCC	HBCC_82011_FTX_H1	11776	5789	27758	83	7.98635

HBCC	HBCC_82011_FTX_H2	11138	6527	26444	100	8.78118
HBCC	HBCC_82012_FTX_H1	13050	9093.5	25401	138	14.8463
HBCC	HBCC_82012_FTX_H2	13335	9951.5	24239	94	8.62462
HBCC	HBCC_82013_FTX_H1	9612	5234.5	20148	148	11.248
HBCC	HBCC_82013_FTX_H2	9928	5608.5	19518	134	8.6927
HBCC	HBCC_82014_FTX_H1	10969	6007.5	24031	158	11.6278
HBCC	HBCC_82014_FTX_H2	12328	7562	26587	151	13.4765
HBCC	HBCC_82015_FTX_H1	12818	6521.5	26577	108	10.0638
HBCC	HBCC_82015_FTX_H2	13827	8388.5	29503	96	7.40214
**HBCC**	**HBCC_82016_FTX_H1**	**40247**	**42930**	**50100**	**5**	**2.59152**
**HBCC**	**HBCC_82016_FTX_H2**	**30035**	**30035**	**30274**	**2**	**1.73825**
HBCC	HBCC_82017_FTX_H1	17255	15540	30023	74	10.9725
HBCC	HBCC_82017_FTX_H2	19353	16848	36269	66	9.34342
HBCC	HBCC_82018_FTX_H1	9007	1751.5	29819	108	7.31899
HBCC	HBCC_82018_FTX_H2	9882	2644	31455	109	8.51317
HBCC	HBCC_82019_FTX_H1	9704	5379	17864	173	16.2523
HBCC	HBCC_82019_FTX_H2	9895	6114	19251	143	11.5249
HBCC	HBCC_82020_FTX_H1	13755	6898.5	29829	102	10.0662
HBCC	HBCC_82020_FTX_H2	11453	4775.5	29837	114	10.081
HBCC	HBCC_82021_FTX_H1	16199	12274.5	29813	98	12.362
HBCC	HBCC_82021_FTX_H2	17307	18756	29245	74	10.6615
HBCC	HBCC_82022_FTX_H1	15258	13330.5	25227	68	10.1417
HBCC	HBCC_82022_FTX_H2	16902	13224	29990	79	10.288
HBCC	HBCC_82023_FTX_H1	11337	6354	23410	104	9.07401
HBCC	HBCC_82023_FTX_H2	12895	7846	24920	105	11.3934
HBCC	HBCC_82024_FTX_H1	8965	5445	17370	116	7.62177
HBCC	HBCC_82024_FTX_H2	7735	3985	16885	165	14.0957
HBCC	HBCC_82025_FTX_H1	11288	7051	21478	107	11.183
HBCC	HBCC_82025_FTX_H2	13326	9849	23977	129	15.1175
HBCC	HBCC_82026_FTX_H1	12948	5906	28784	101	9.84486
HBCC	HBCC_82026_FTX_H2	15315	9770	28377	125	14.2391
HBCC	HBCC_82027_FTX_H1	17474	15801	28374	83	10.1398
HBCC	HBCC_82027_FTX_H2	17394	13890	33248	81	11.0888
HBCC	HBCC_82028_FTX_H1	24021	24002	31927	51	10.1601
HBCC	HBCC_82028_FTX_H2	25140	25806	36948	57	9.67177
HBCC	HBCC_82029_FTX_H1	17705	14171.5	30749	84	10.3019
HBCC	HBCC_82029_FTX_H2	18861	17483	29403	93	11.8072
HBCC	HBCC_82030_FTX_H1	15590	14421	29102	74	8.5451
HBCC	HBCC_82030_FTX_H2	17348	14556.5	31726	72	9.17596

HBCC	HBCC_82031_FTX_H1	17005	14957.5	26549	72	8.43948
HBCC	HBCC_82031_FTX_H2	18044	15712	30239	75	10.2125
HBCC	HBCC_82032_FTX_H1	20738	22120	31732	65	10.7405
HBCC	HBCC_82032_FTX_H2	20098	17379	29559	42	6.58787
HBCC	HBCC_82033_FTX_H1	11183	8355	22983	151	13.7346
HBCC	HBCC_82033_FTX_H2	12010	8704	22156	143	13.0909
HBCC	HBCC_82034_FTX_H1	11669	7191	23315	105	10.1569
HBCC	HBCC_82034_FTX_H2	10535	6262	20570	96	7.49028
HBCC	HBCC_82035_FTX_H1	15373	10487	27358	89	9.68911
HBCC	HBCC_82035_FTX_H2	16017	12110	26972	87	11.6625
HBCC	HBCC_82036_FTX_H1	10135	6032	18468	90	8.76148
HBCC	HBCC_82036_FTX_H2	8112	3216	22779	58	3.77658
HBCC	HBCC_82038_FTX_H1	7700	4586	14337	229	13.2693
HBCC	HBCC_82038_FTX_H2	7873	4725	15309	219	13.3964
HBCC	HBCC_82039_FTX_H1	8514	3126	20654	131	9.95247
HBCC	HBCC_82039_FTX_H2	8558	3320	19600	117	8.34618
HBCC	HBCC_82040_FTX_H1	12282	6235	26264	90	8.37307
HBCC	HBCC_82040_FTX_H2	13425	8426.5	26798	94	10.3773
HBCC	HBCC_82042_FTX_H1	17525	15984	26167	81	11.2581
HBCC	HBCC_82042_FTX_H2	15131	12157	25416	83	8.92197
HBCC	HBCC_82043_FTX_H1	11200	6919	21949	150	11.2007
HBCC	HBCC_82043_FTX_H2	10363	4595	23952	134	9.45819
HBCC	HBCC_82044_FTX_H1	10487	4903.5	26431	134	11.2494
HBCC	HBCC_82044_FTX_H2	11131	7921.5	24764	142	11.1625
HBCC	HBCC_82045_FTX_H1	19225	16606	31818	90	12.5025
HBCC	HBCC_82045_FTX_H2	12413	3514	31354	109	11.0838
HBCC	HBCC_82046_FTX_H1	17833	12226.5	30844	98	12.7492
HBCC	HBCC_82046_FTX_H2	23722	25735	35133	77	12.6591
HBCC	HBCC_82048_FTX_H1	18109	15715	30068	81	10.8223
HBCC	HBCC_82048_FTX_H2	14938	14477	24576	83	9.31311
HBCC	HBCC_82050_FTX_H1	15802	10993.5	29965	72	8.66966
HBCC	HBCC_82050_FTX_H2	17232	15866	27464	63	9.85042
HBCC	HBCC_82051_FTX_H1	13464	10385	23654	138	11.6601
HBCC	HBCC_82051_FTX_H2	15315	12444.5	27137	158	17.2567
HBCC	HBCC_82052_FTX_H1	9431	6183.5	20334	68	6.21083
HBCC	HBCC_82052_FTX_H2	10035	5065	21655	77	5.8544
HBCC	HBCC_82053_FTX_H1	19198	18060.5	30402	86	12.25
HBCC	HBCC_82053_FTX_H2	22581	24695	33314	66	10.3078
HBCC	HBCC_82054_FTX_H1	16176	10488	35362	88	10.5471

HBCC	HBCC_82054_FTX_H2	17127	8550	35521	63	7.70821
HBCC	HBCC_82055_FTX_H1	19712	20287	30558	52	8.05796
HBCC	HBCC_82055_FTX_H2	15858	11519	32555	75	8.63674
HBCC	HBCC_82056_FTX_H1	14670	13360.5	22767	80	9.67254
HBCC	HBCC_82056_FTX_H2	14483	11554	23341	79	8.70578
HBCC	HBCC_82057_FTX_H1	26294	27182	37684	42	9.57004
HBCC	HBCC_82057_FTX_H2	17288	14299	30548	34	6.59473
HBCC	HBCC_82058_FTX_H1	19014	17619	28873	57	8.42055
HBCC	HBCC_82058_FTX_H2	21446	24961	31966	45	7.23156
HBCC	HBCC_82059_FTX_H1	21975	24729	28424	39	6.23434
HBCC	HBCC_82059_FTX_H2	18328	18288	28286	47	6.94743
HBCC	HBCC_82061_FTX_H1	12406	6706.5	22771	128	11.4566
HBCC	HBCC_82061_FTX_H2	11693	7038	26570	108	9.83012
HBCC	HBCC_82062_FTX_H1	15443	12089	27444	73	8.38505
HBCC	HBCC_82062_FTX_H2	17963	15054.5	29544	72	10.3551
HBCC	HBCC_82063_FTX_H1	7028	3676	18461	221	12.5515
HBCC	HBCC_82063_FTX_H2	6393	3368	16020	204	13.8417
HBCC	HBCC_82064_FTX_H1	7810	4964.5	12661	224	13.5693
HBCC	HBCC_82064_FTX_H2	7503	4785	12764	273	16.8494
HBCC	HBCC_82065_FTX_H1	6355	3630.5	12254	248	13.7367
HBCC	HBCC_82065_FTX_H2	6778	4148	12692	212	14.409
HBCC	HBCC_82066_FTX_H1	10458	10066.5	18490	80	8.5753
HBCC	HBCC_82066_FTX_H2	10334	9726	15462	54	5.56393
HBCC	HBCC_82067_FTX_H1	23654	28641	37162	33	6.12065
HBCC	HBCC_82067_FTX_H2	22676	22442	29646	25	5.18313
HBCC	HBCC_82068_FTX_H1	14691	12852.5	27351	84	9.17862
HBCC	HBCC_82068_FTX_H2	18353	16310	28095	101	12.8004
HBCC	HBCC_82069_FTX_H1	16820	17010	26324	58	8.88304
HBCC	HBCC_82069_FTX_H2	8868	3816.5	18401	64	6.48337
HBCC	HBCC_82074_FTX_H1	12753	7252	26988	110	12.0793
HBCC	HBCC_82074_FTX_H2	11316	5202	25880	104	9.17536
HBCC	HBCC_82075_FTX_H1	15519	12901	28375	115	13.7425
HBCC	HBCC_82075_FTX_H2	14294	10293	24754	115	11.3899
HBCC	HBCC_82076_FTX_H1	20071	16343	35388	75	10.6961
HBCC	HBCC_82076_FTX_H2	18244	15361	28720	84	10.9617
	Avg	13859.87786	10717.01718	25623.62595	114.2748092	10.63284111
	Stdv	5059.861412	6816.009052	5994.2115	61.33291128	2.614708622

Table S4: Comparison of allele-specific and allele-averaged CpG sites in the NABEC and HBCC cohorts. Allele-specific CpG sites are shown on the left and allele-averaged CpG sites on the right. N denotes the number of samples included in each model; beta represents the estimated effect size; SE is the standard error; and pval_adj is the BH-FDR–corrected p-value. The pva_adj values of statistically significant results are bolded.

			Allele-specific methylation results					Allele-averaged methylation results				
Cohort	CpG site	Contrast	N_AS	beta_AS	SE_AS	pval_AS	BH-FDR corrected pval_AS	N_AA	beta_AA	SE_AA	pval_AA	BH-FDR corrected pval AA
NABEC	cpg_chr19_44901266	e4	351	-5.49	1.38	9.03E-05	2.81E-02	187	-3.00	1.02	3.83E-03	7.32E-01
NABEC	cpg_chr19_44914329	e2	361	8.32	1.96	2.81E-05	1.46E-02	187	5.64	1.32	3.30E-05	**2.57E-02**
NABEC	cpg_chr19_44914329	e4	361	8.07	2.08	1.25E-04	3.23E-02	187	2.78	1.49	6.45E-02	9.28E-01
NABEC	cpg_chr19_44914361	e2	340	-8.91	2.2	6.27E-05	2.44E-02	187	-2.60	1.49	8.30E-02	9.54E-01
NABEC	cpg_chr19_44917921	e4	363	-7.96	1.77	9.67E-06	7.52E-03	187	-2.99	1.28	2.02E-02	8.96E-01
NABEC	cpg_chr19_44917959	e4	357	-7.51	1.89	8.51E-05	2.81E-02	187	-3.69	1.44	1.13E-02	7.85E-01
NABEC	cpg_chr19_44917997	e4	364	-29.74	2.13	3.15E-35	4.90E-32	187	-16.04	1.51	2.13E-20	**3.30E-17**
NABEC	cpg_chr19_44918064	e4	360	-8.79	2.12	4.24E-05	2.20E-02	187	-3.73	1.51	1.49E-02	8.77E-01
NABEC	cpg_chr19_44921919	e2	344	-13.51	2.25	5.45E-09	4.24E-06	187	-9.96	1.42	6.06E-11	**9.42E-08**
HBCC	cpg_chr19_44890618	rs769455[T]	238	1.15	0.29	1.01E-04	1.71E-02	123	0	0	8.73E-01	9.61E-01
HBCC	cpg_chr19_44890901	rs769455[T]	249	2.29	0.6	1.94E-04	2.47E-02	124	0	0	8.23E-01	9.61E-01
HBCC	cpg_chr19_44896082	e4	246	-4.56	0.97	4.36E-06	6.66E-03	124	-2.19	0.77	5.17E-03	9.61E-01
HBCC	cpg_chr19_44896114	rs769455[T]	248	-4.93	1.29	1.64E-04	2.37E-02	124	0	0	1.34E-03	5.99E-02
HBCC	cpg_chr19_44896243	rs769455[T]	249	-12.5	3.08	6.72E-05	1.66E-02	124	0	0	7.88E-01	9.53E-01
HBCC	cpg_chr19_44899850	rs769455[T]	248	-10.11	2.55	9.65E-05	1.71E-02	124	0	0	2.66E-01	6.82E-01
HBCC	cpg_chr19_44904817	rs769455[T]	245	-30.24	5.49	1.03E-07	5.26E-05	124	0	0	9.49E-01	9.85E-01
HBCC	cpg_chr19_44915122	rs769455[T]	250	1.64	0.35	4.07E-06	1.56E-03	124	0	0	1.47E-01	5.33E-01
HBCC	cpg_chr19_44915466	rs769455[T]	252	2.87	0.7	6.23E-05	1.66E-02	124	0	0	2.14E-01	6.23E-01
HBCC	cpg_chr19_44931155	rs769455[T]	249	-9.01	2.23	7.58E-05	1.66E-02	124	0	0	7.11E-01	9.36E-01
HBCC	cpg_chr19_44941331	rs769455[T]	251	-5.4	1.41	1.70E-04	2.37E-02	124	0	0	8.29E-01	9.61E-01

Table S5: methQTL results from linear regression analyses testing the association between APOE ε2 and ε4 alleles and CpG methylation levels across the TOMM40, APOE, and APOC1 genes.

Cohort	Gene	Allele/Variant	N	beta	SE	pval	BH-FDR corrected pval
NABEC	*TOMM40*	e2	201	-0.01	0.01	1.80E-01	5.40E-01
NABEC	*TOMM40*	e4	201	0.01	0.01	3.32E-01	7.18E-01
NABEC	*APOE*	e4	201	0	0.01	7.18E-01	7.18E-01
NABEC	*APOC1*	e4	201	0.01	0.02	5.53E-01	7.18E-01
NABEC	*APOE*	e2	201	0.01	0.01	4.89E-01	7.33E-01
NABEC	*APOC1*	e2	201	0	0.02	9.27E-01	9.27E-01
HBCC	*APOC1*	e2	71	0.08	0.04	5.76E-02	1.73E-01
HBCC	*APOE*	e2	71	-0.05	0.04	1.66E-01	2.49E-01
HBCC	*TOMM40*	e2	71	0.03	0.03	2.57E-01	2.57E-01
HBCC	*TOMM40*	e4	71	0.01	0.02	4.66E-01	4.66E-01
HBCC	*APOE*	e4	71	0.03	0.02	1.70E-01	4.66E-01
HBCC	*APOC1*	e4	71	0.02	0.02	3.88E-01	4.66E-01
HBCC	*TOMM40*	rs769455[T]	71	0.01	0.04	8.07E-01	8.07E-01
HBCC	*APOE*	rs769455[T]	71	0.04	0.07	5.77E-01	8.07E-01
HBCC	*APOC1*	rs769455[T]	71	-0.03	0.07	6.45E-01	8.07E-01

Table S6: eQTM results were derived from linear regression models testing associations between CpG methylation and expression of TOMM40, APOE, and APOC1, while adjusting for APOE ε2, ε4, and rs769455[T] dosages.

Cohort	Gene	CpG	N	beta	SE	pval	BH-FDR corrected pval
NABEC	*TOMM40*	cpg_chr19_44901266	186	0.00044	0.00069	0.52369	0.88854
NABEC	*TOMM40*	cpg_chr19_44914329	187	0.00026	0.00049	0.58852	0.90536
NABEC	*TOMM40*	cpg_chr19_44914361	185	-0.00007	0.00043	0.87211	0.96047
NABEC	*TOMM40*	cpg_chr19_44917921	187	0.00014	0.00058	0.81171	0.94650
NABEC	*TOMM40*	cpg_chr19_44917959	187	0.00014	0.00049	0.78435	0.94217
NABEC	*TOMM40*	cpg_chr19_44917997	187	0.00011	0.00047	0.81882	0.95052
NABEC	*TOMM40*	cpg_chr19_44918064	187	0.00026	0.00047	0.57231	0.89612
NABEC	*TOMM40*	cpg_chr19_44921919	187	0.00020	0.00045	0.66285	0.92459
NABEC	*APOE*	cpg_chr19_44901266	186	-0.00075	0.00113	0.50654	0.94270
NABEC	*APOE*	cpg_chr19_44914329	187	-0.00013	0.00080	0.87418	0.98785

NABEC	*APOE*	cpg_chr19_44914361	185	-0.00009	0.00070	0.89422	0.98785
NABEC	*APOE*	cpg_chr19_44917921	187	0.00204	0.00093	0.02999	0.92535
NABEC	*APOE*	cpg_chr19_44917959	187	-0.00068	0.00081	0.40219	0.94270
NABEC	*APOE*	cpg_chr19_44917997	187	0.00061	0.00077	0.43067	0.94270
NABEC	*APOE*	cpg_chr19_44918064	187	-0.00032	0.00076	0.67794	0.97716
NABEC	*APOE*	cpg_chr19_44921919	187	0.00080	0.00073	0.27474	0.94270
NABEC	*APOC1*	cpg_chr19_44901266	186	-0.00144	0.00132	0.27902	0.99723
NABEC	*APOC1*	cpg_chr19_44914329	187	-0.00076	0.00095	0.42545	0.99723
NABEC	*APOC1*	cpg_chr19_44914361	185	0.00047	0.00082	0.57181	0.99723
NABEC	*APOC1*	cpg_chr19_44917921	187	0.00179	0.00111	0.10900	0.99723
NABEC	*APOC1*	cpg_chr19_44917959	187	-0.00002	0.00096	0.98741	0.99723
NABEC	*APOC1*	cpg_chr19_44917997	187	0.00128	0.00090	0.15860	0.99723
NABEC	*APOC1*	cpg_chr19_44918064	187	0.00084	0.00090	0.34969	0.99723
NABEC	*APOC1*	cpg_chr19_44921919	187	-0.00017	0.00087	0.84255	0.99723
HBCC	*TOMM40*	cpg_chr19_44904817	55	0.00042	0.00102	0.68241	0.98864
HBCC	*TOMM40*	cpg_chr19_44906193	55	0.00017	0.00595	0.97686	0.98864
HBCC	*TOMM40*	cpg_chr19_44915122	55	-0.01191	0.01188	0.32242	0.98864
HBCC	*TOMM40*	cpg_chr19_44915466	55	0.00458	0.00664	0.49449	0.98864
HBCC	*TOMM40*	cpg_chr19_44921967	54	0.00202	0.00664	0.76233	0.98864
HBCC	*TOMM40*	cpg_chr19_44931155	55	-0.00331	0.00294	0.26592	0.98864
HBCC	*TOMM40*	cpg_chr19_44941331	55	-0.00884	0.00551	0.11702	0.98864
HBCC	*APOE*	cpg_chr19_44904817	55	0.00017	0.00157	0.91493	0.99993
HBCC	*APOE*	cpg_chr19_44906193	55	0.01404	0.00884	0.12068	0.89509
HBCC	*APOE*	cpg_chr19_44915122	55	-0.01648	0.01826	0.37262	0.99993
HBCC	*APOE*	cpg_chr19_44915466	55	0.00100	0.01025	0.92240	0.99993
HBCC	*APOE*	cpg_chr19_44921967	54	0.01056	0.00974	0.28549	0.99993
HBCC	*APOE*	cpg_chr19_44931155	55	-0.00162	0.00457	0.72429	0.99993
HBCC	*APOE*	cpg_chr19_44941331	55	-0.01376	0.00844	0.11140	0.88643
HBCC	*APOC1*	cpg_chr19_44904817	55	-0.00141	0.00136	0.30894	0.99463
HBCC	*APOC1*	cpg_chr19_44906193	55	0.00470	0.00803	0.56150	0.99463
HBCC	*APOC1*	cpg_chr19_44915122	55	-0.00201	0.01629	0.90243	0.99997
HBCC	*APOC1*	cpg_chr19_44915466	55	-0.00825	0.00895	0.36205	0.99463
HBCC	*APOC1*	cpg_chr19_44921967	54	0.01129	0.00881	0.20799	0.99463
HBCC	*APOC1*	cpg_chr19_44931155	55	-0.00206	0.00403	0.61140	0.99463
HBCC	*APOC1*	cpg_chr19_44941331	55	-0.00833	0.00759	0.27923	0.99463

Table S7: Summary of estimated cell-type proportions for NABEC and HBCC cohorts. Cell-type deconvolution of single-nucleus RNA-seq data from adjacent cortical tissue reveals the distribution of six major brain cell populations—astrocytes (Astro), excitatory neurons (ExN), inhibitory neurons (InN), microglia (MG), oligodendrocyte precursor cells (OPC), and vascular cells (VC). Mean percentages, standard deviations (SD), medians, and ranges are reported for NABEC (N = 168) and HBCC (N = 127) samples.

**NABEC cohort**						
	**mean**	**sd**	**median**	**min**	**max**	**N**
**Astro**	7.31288	6.14388	6.44964	0.17401	43.52159	168
**ExN**	23.21822	14.43807	22.20385	0.43026	58.70525	168
**InN**	13.31704	8.23251	12.29973	0.23595	46.92308	168
**MG**	6.01587	2.65614	5.63598	0.50324	17.37874	168
**OPC**	5.74083	2.63018	5.02703	1.53846	17.57085	168
**Oligo**	43.44590	23.01090	41.42910	2.54650	90.00510	168
**VC**	0.94926	0.96839	0.65656	0.00000	5.37356	168
**HBCC cohort**						
	**mean**	**sd**	**median**	**min**	**max**	**N**
**Astro**	12.72941	10.25036	10.70163	0.20721	77.66497	127
**ExN**	28.18255	15.39333	28.70021	0.00000	68.06723	127
**InN**	14.87697	7.30016	14.70054	0.46671	40.37308	127
**MG**	5.22684	3.61455	4.44184	0.05959	17.89048	127
**OPC**	6.49564	2.33344	6.13748	1.80072	15.45828	127
**Oligo**	31.08955	21.28387	26.81664	1.08043	90.42802	127
**VC**	1.39905	1.03247	1.05235	0.00000	5.13994	127
